# Form and Function in Mobulids: A Comparative Analysis of Filter Morphology With Bioinspiration Applications

**DOI:** 10.1093/icb/icaf142

**Published:** 2025-08-01

**Authors:** J B Teeple, S R Kahane-Rapport, K E Cohen, L Hamann, J A Strother, E W M Paig-Tran

**Affiliations:** Biological Sciences, California State University Fullerton, 800 State College Blvd. Fullerton, CA 92831, USA; Biological Sciences, California State University Fullerton, 800 State College Blvd. Fullerton, CA 92831, USA; Biological Sciences, Old Dominion University, 5115 Hampton Boulevard, Norfolk, VA 23529, USA; Biological Sciences, California State University Fullerton, 800 State College Blvd. Fullerton, CA 92831, USA; University of Washington, Friday Harbor Laboratories, 620 University Way, Friday Harbor, WA 98250, USA; Department of Biology, University of Florida, Whitney Marine Laboratory for Marine Bioscience, 9505 North Ocean Shore Boulevard, St. Augustine, FL 32080, USA; Bonn Institute for Organismic Biology, Section 2, Animal Diversity, University of Bonn, An der Immenburg 1, 53121 Bonn, Germany; Department of Biology, University of Florida, Whitney Marine Laboratory for Marine Bioscience, 9505 North Ocean Shore Boulevard, St. Augustine, FL 32080, USA; Biological Sciences, California State University Fullerton, 800 State College Blvd. Fullerton, CA 92831, USA

## Abstract

Mobulas (manta and devil rays) are large-scale ram filter feeders that separate planktonic food particles from large volumes of water with minimal clogging. This contrasts with most human-made filters that can suffer from problematic clogging requiring additional mechanisms for clearing blocked surfaces and maintaining performance. Prior studies have shown that mobulas employ a unique mechanism referred to as ricochet separation to filter feed, whereby captive vortices in filter pores cause particles to bounce off the filter surfaces and away from the filter pores. This mechanism enables the filtration of particles smaller than the pore size and reduced clogging. However, few studies have examined how the morphology of the filtering structure varies across the diversity of mobulid species, and little is known about how this variation may impact filtration efficiency or prey selectivity. This study conducts a systematic investigation of the gross morphology of the filtering structure in seven mobilid species using a combination of computed tomography and macro photography. Examination of filter anatomy suggests that some features are highly variable while others are well-conserved across species. In particular, a reconstruction of the phylogenetically corrected morphospaces indicated that the primary pore dimensions of the filter lobes are a major driver of morphological variation across species. Additionally, inspection of the gross anatomy revealed a pronounced asymmetry in the anterior and posterior filter plates of each gill arch. This asymmetry suggests that water may impinge on the filtering structures at different angles than has previously been speculated. Here, the functional ramifications of the observed morphological variations were interpreted using recent modeling studies. Most mobulid fishes have a filter morphology that should be capable of high filtration efficiency and low hydrodynamic resistance, but may also be sensitive to flow conditions. A deeper understanding of the mechanics of filter-feeding in mobulid fishes would generate needed insights into the ecology of these species and could provide a firmer framework for the development of bioinspired filtration systems. These findings highlight the value of integrating detailed anatomical studies into bioinspired design efforts and pave the way for the development of bioinspired filter systems with improved performance.

## Introduction

**Fig. 1:**
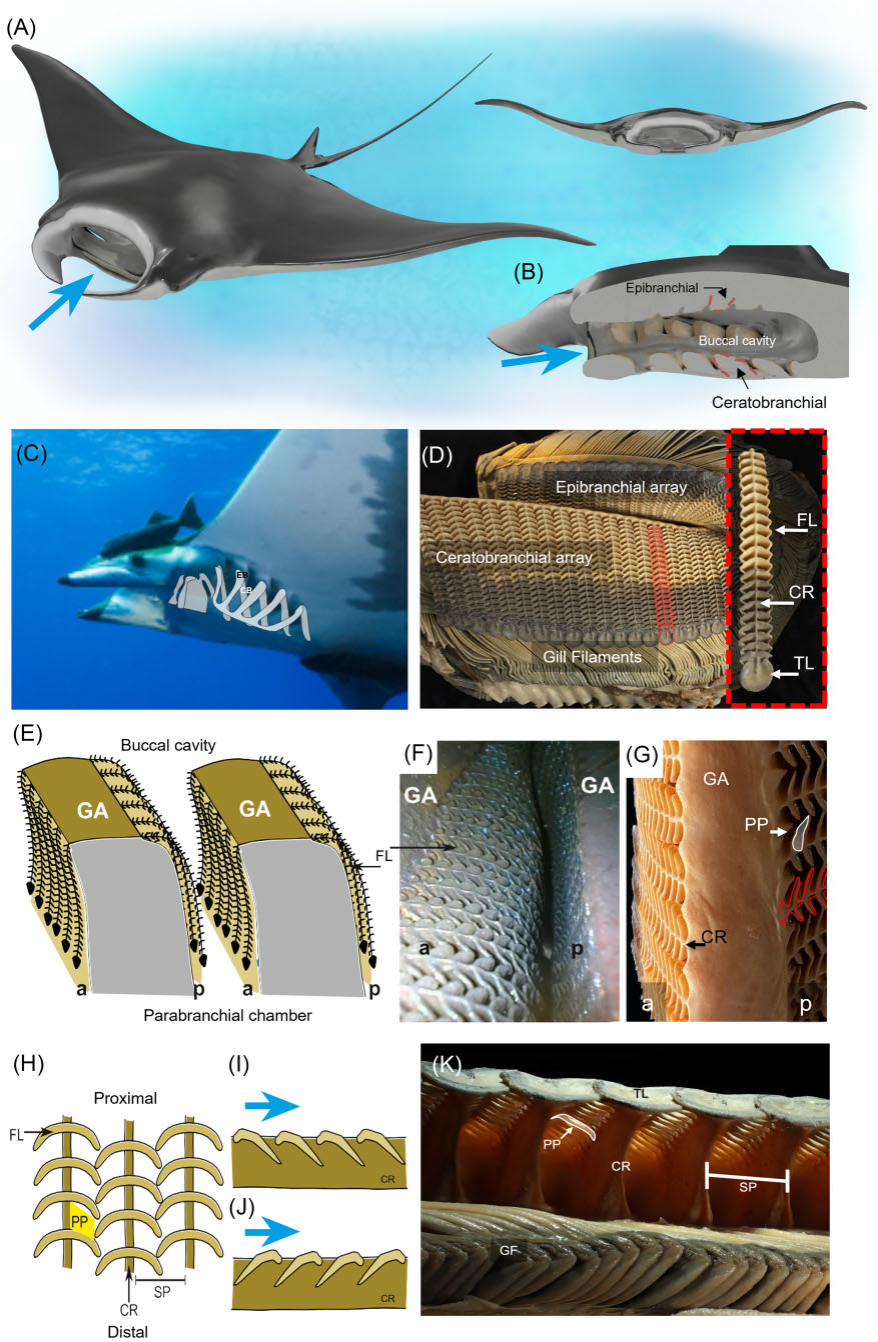
Generalized gross morphology of Mobulids. (A) An illustration of a feeding *M. birostris*, swimming open-mouthed; large arrow represents the oncoming water flow. Image courtesy of the Digital Life Project at the University of Massachusetts at Amherst. (B) A parasagittal plane view of the buccal cavity, with the dorsal epibranchial and ventral ceratobranchial arches identified. (C) A live *M. tarapacana* with an overlaid schematic of the branchial basket. Epibranchial (EB) and ceratobranchial (CB) arches identified. (D) A dissected gill arch displaying the epibranchial and ceratobranchial arrays of filter elements and gill filaments, with the inset highlighting specific anatomical features of a single filter plate (outlined on arch and enlarged in inset), including the filter lobes (FL), central raphe (CR), and terminal lobe (TL). (E) Schematic drawing of a cross-section through the parasagittal plane showing the anterior (a) and posterior (p) filter lobes on the gill arches (GA). Schematic based on observations from CT scans and fixed specimens, like the real image shown in (F). (F) Lateral view of the gill arches inside the buccal cavity of a preserved *M. hypostoma*, captured using an endoscope. Anterior plate shows the shelf-like ledge along the filter prior to changing angles and dipping into the interbranchial space. (G) A close-up of the filter array reveals overall filter anatomy, including the gill arch (GA), central raphe (CR), and primary pore (PP, outlined in white); ascending filter lobes are outlined in red. (H) Schematic illustrating the positioning and structure of ascending filter plates and central raphe (CR). Primary pore highlighted and secondary pore (SP) indicated between central raphes. (I) Schematic showing how incoming water (large arrow) would flow over filter lobes on a central raphe (CR) in the spoiler orientation and (J) in the wing orientation. (K) A medial view of the filter plates showing the underside of the plates, the connection of the central raphe (CR) to the arch, the gill filaments (GF), and the spacing of the secondary pores (SP). The primary pore is marked by a solid outline with semi-transparent fill in (G) and (K).

Mobulid fishes are large pelagic filter-feeding rays that are broadly distributed across warm and temperate seas (Couturier et al. 2012). Inside their buccopharynx, these rays possess five pairs of branchial arches that support the branchial filters and the paired rows of respiratory gill filaments (each filament row is a hemibranch; two rows of filaments constitute a holobranch) (Wilson and Laurent 2002). The branchial arches are located posterior to the hyoid arch and bilaterally within the pharyngeal cavity. Each arch is composed of a series of skeletal elements including (from dorsal to ventral): the pharyngobranchial, epibranchial, ceratobranchial, hypobranchial, and basibranchial. The filter elements in fishes are bony, cartilaginous, or keratinized processes that project from the branchial arch (along the epibranchials and/or ceratobranchials) and are used to separate prey from water. The branchial filters in mobulid fishes differ markedly from the elongated gill rakers that occur in many filter-feeding bony fishes (Fig. 1B). In bony fishes, the filters are called gill rakers and are typically located along the medial edge of the branchial arches and project toward the buccal cavity. In contrast, the filters of mobulid fishes are shifted to both the anterior and posterior surfaces of the branchial arches along the epibranchials and ceratobranchials (Fig. 1B, E). The filters are flattened rather than elongate and have been referred to as “gill sieves” (Bigelow and Schroeder 1953) or “branchial filter plates” (Notarbartolo-Di-Sciara 1987) as a nod to this flattened, pad-like morphology. There are four arrays of branchial filters on each arch: (1) the ceratobranchial anterior, (2) the ceratobranchial posterior, (3) the epibranchial anterior, and (4) the epibranchial posterior; each array of branchial filters consists of many rows of individual filter plates. Each individual filter plate consists of repeating, paired, leaf-like filter lobes, connected via a central raphe to the branchial arch- similar to a fern whose paired leaves or pinna are connected by the central petiole. The filtering lobes consist of a cartilaginous core covered by a keratinous epithelium (Paig‐Tran and Summers 2014). The spaces separating the ascending filter lobes on either side of the central raphe are termed primary pores (Fig. 1D, H) and feed into a larger channel referred to as the secondary pore, formed from the space between two adjacent central raphes as they connect to the branchial arch (Fig. 1K; Paig‐Tran et al. 2013). The distalmost edge of the filter consists of a singular, rounded terminal lobe located nearest the interbranchial septum, where water is funneled over the respiratory gill filaments (Fig. 1B, K). The interbranchial septum separates the gill hemibranchs and is composed of connective tissue that runs from the branchial arch to the outer body wall. The spaces between adjacent interbranchial septa form the parabranchial spaces that open externally as gill slits (Hughes and Morgan 1973; Wilson and Laurent 2002).

Mobulid rays exhibit remarkable diversity in their filter morphology. The surface of the filter lobes range from smooth (*Mobula kuhlii, Mobula mobular, Mobula munkiana*) to covered with conical denticles (*Mobula birostris, Mobula hypostoma, Mobula tarapacana, Mobula thurstoni*; Paig-Tran et al. 2013). The filter lobes range from heart shaped (*M. tarapacana, M. thurstoni*) to broadened and rectangularly shaped (*M. birostris, M. kuhlii, M. mobular*; Paig-Tran et al. 2013). The shape of the filter lobes can vary along an individual filter plate moving from the base (proximal) toward the distal lobes. For example, both *M. munkiana* and *M. hypostoma* are described as having heart shaped lobes near the filter plate base changing to broadened lobes near the terminal lobe (Paig-Tran et al. 2013). In two cases, filter lobes from adjacent individual plates fuse together (*M. birostris, M. tarapacana*) via connective tissues, while in others, the filter plates may be barely touching or completely separated from lobes on adjacent plates (*M. mobular, M. kuhlii*). Some filter lobes contain mucosal cells (*M. tarapacana, M. munkiana, M. kuhlii*) and/or cilia (*M. kuhlii, M. tarapacana*; Paig‐Tran and Summers 2014).

Mobulas were once widely believed to separate zooplankton from sea water using either hydrosol or simple sieve filtration (Smith 1943; Ormond and Edwards 1987; Albert 2007). Hydrosol filtration captures food particles along adhesive surfaces. In sieve filtration, particle-laden fluid passes through a porous filter membrane; particles larger than the pore are trapped on the filter surface, while water and smaller particles travel through the pores (Rubenstein and Koehl 1977). However, both hydrosol and sieve filters tend to clog quickly and require a secondary mechanism for removal of clogged material. Although some mobulas have been observed “coughing” to release particles from their buccal cavity (Klimley et al. 2024), mobulas do not have fleshy tongues or other obvious anatomical features that could serve this function; therefore, sieving has been ruled out as a likely mechanism for filtration in the mobulas as they rarely, if ever, suffer from clogging. A histological survey of mobula filters showed that only three species had mucosal cells present along their filters, suggesting hydrosol filtration was also an unlikely candidate for filtration across the mobulids (Paig‐Tran and Summers 2014). Cross-flow filtration emerged as a possible mechanism for mobula filtration after it was proposed as the mechanism in some bony fishes and possibly in whale sharks (Motta et al. 2010). In cross-flow filtration, water and food particles flow approximately parallel to the gill rakers/filter elements (Smith and Sanderson 2008; Hamann et al. 2023; Kahane-Rapport and Paig-Tran 2023; Sanderson 2024). Food particles larger than the pore of the filter are retained and then swept toward the esophagus via a tangential flow (Sanderson et al. 2001). While this mechanism accounts for the reduced clogging seen in the mobulas, it is an unlikely mechanism as this would limit targeted prey type to only those larger than the filter pore (Paig-Tran et al. 2011). So, how do mobulas filter their planktonic prey?

Mobulid rays are large, fast-moving animals and *in situ* studies are experimentally difficult. As such, physical models and computational studies have been key to investigating the mechanics of filtration (Divi et al. 2018; Mao et al. 2024; Kahane-Rapport et al. 2025). Computational fluid dynamics (CFD) models based on the morphology of oceanic manta rays (*M. birostris*) and sickle fin devil rays (*M. tarapacana*) indicated that these rays use a previously undescribed mechanism of filtration that was termed “ricochet separation” (Divi et al. 2018). In this filtration mechanism, particle laden water flows over the filter lobes. Tangential flow induces flow separation downstream of the leading edge of the filter lobe, resulting in the formation of captive vortices within the primary pore. This flow pattern causes streamlines traveling through the filter pores to pass very near to the filter surfaces. Solid particles following these streamlines contact the filter surfaces and are redirected away (or ricochet) from the filter into streamlines that pass over the filter pore—often bouncing from one filter lobe to the next. This process was found to enable the filtration of particles as small as ⅕ the pore size of the filter with minimal clogging (Divi et al. 2018).

The relationship between the morphology of the filter and the filter performance has been explored though physical and theoretical modeling. Physical and CFD models based on *M. tarapacana* filters indicated that filtration efficiency is largely insensitive to the pore size (Kahane-Rapport et al. 2025). In contrast, the angle of the filter lobes relative to the filter plate was found to have pronounced effects on the filter performance, with steeper angles tending to improve filtration of smaller particle sizes. The plates and lobes may be positioned such that the leading edge of the lobes points against the direction of flow (wing-like orientation; Fig. 1I), or they may be oriented such that the leading edge of the lobes are aligned with the direction of flow (spoiler-like orientation; Fig. 1J). Filter plates in which the pores were directed toward the freestream flow (wing-like orientation) tended to display lower hydrodynamic resistance, while filter plates in which the pores were directed away from the freestream flow (spoiler-like orientation) exhibited higher filtration efficiency (Kahane-Rapport et al. 2025). These results are principally consistent with a recent analytical model of the flow around the mobulid filter, which describes the flow conditions necessary for flow separation and captive vortex formation (Mao et al. 2024). However, unlike the physical models and computational simulations, this analytical model predicted a strong effect of pore size on the cut-off particle size and additional research may be required to understand this difference.

Advances in our understanding of filter-feeding in mobulid fishes have inspired promising bio-inspired filter designs. At the microfluidic level, manta-derived filter lobes achieved non-clogging, high-throughput microparticle filtration with up to 99% efficiency at processing speeds up to 20 mL/min (Clark and San-Miguel 2021). A U-shaped manta-inspired filter was recently developed to process monodisperse and bi-disperse suspensions and yeast cells at maximum efficiencies up to 97% at 8 mL/min (Hu et al. 2024). Filters based on ricochet separation have also been designed as a way to filter harmful microplastics in coral reef systems, though they have yet to be implemented (Sankrityayan and Biswas 2022). On a larger scale, manta-derived filters have been successfully used to filter algae from water to mitigate harmful algal blooms (Marshall 2019). Furthermore, mobulid filters have inspired numerous student teams to tackle real world problems, such as the Coconets filter designed to intercept point-source ocean plastics, car exhaust filters, and systems designed for purifying drinking water (van der Meer et al. 2019; Chao et al. 2020; Rojas et al. 2022).

While substantial progress has been made in understanding the basic principles of mobulid filtration, many open questions remain. Prior modeling efforts employed simplified geometries, and it is unclear how incorporating the complex 3D shape of the filter lobes will influence the fluid flow and solid-fluid separation. Furthermore, relatively little is known about the morphology and flow patterns within the buccal cavity during feeding, or how these factors influence filtration processes.

We aimed to uncover the morphological variation present in two ways. We first conducted a comprehensive morphometric analysis of mobulid filter structures across multiple species using micro-CT imaging, endoscopic visualization, and phylogenetically informed statistical approaches. By refining anatomical terminology and standardizing measurement protocols, we captured both inter- and intra-species variation in primary and secondary pore dimensions and branchial arch morphology. We then integrated 3D imaging with *in situ* visualization to quantify how filter elements are spatially arranged within the buccal cavity, revealing structural asymmetries along both the anterior–posterior and dorsal–ventral axes.

## Methods

### Specimen sampling

**Fig. 2:**
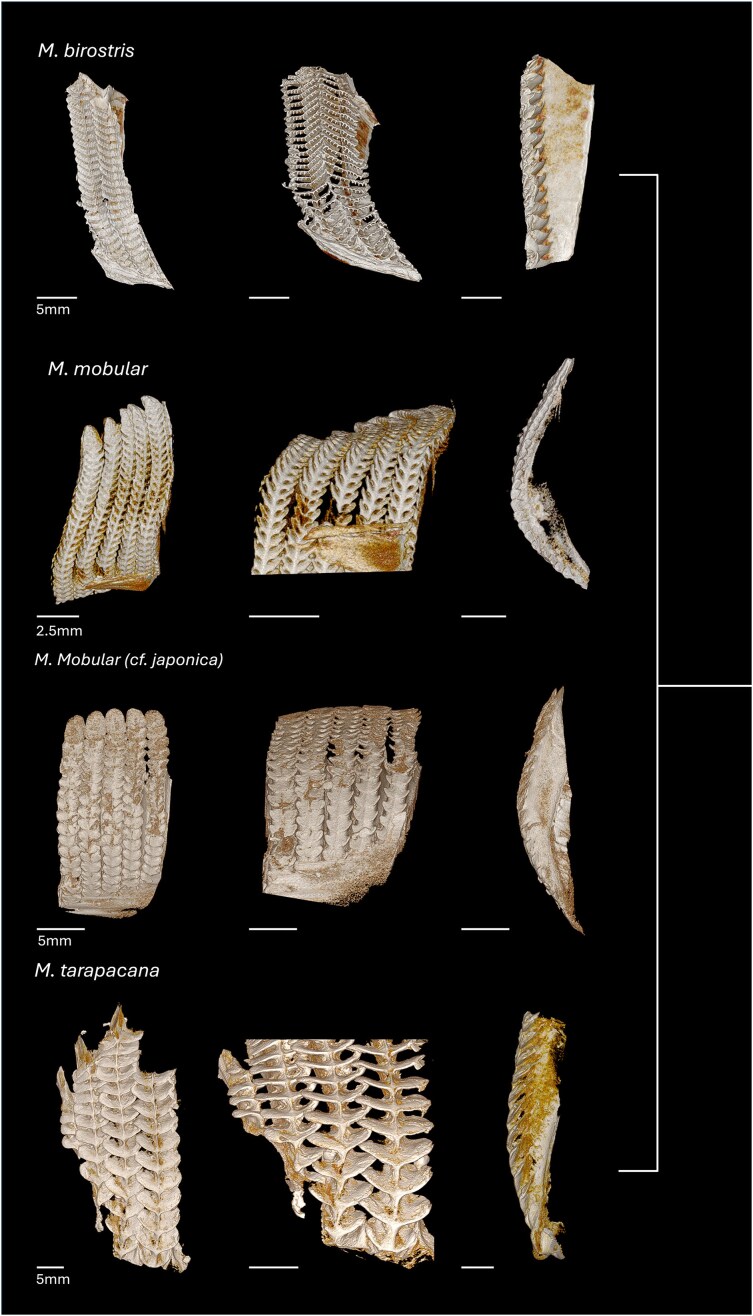
Micro-CT images of filters from four mobulid specimens in the large-bodied clade including M. tarapacana, M. mobular (cf. M. japanica), M. birostris, M. alfredi, and the third putative species M. cs. birostris. The clade is supported by the analysis of mitochondrial genomes, 1000 nuclear exons, and morphologically via the presence of a slit-like spiracle on the dorsal plane of the pectoral fins (White et al. 2018); phylogeny indicated on the right. The first column of images shows the posteroanterior view of the filter plates, illustrating the shape of the filter lobes along the filter plate. The second column shows the dorsoventral view of the filter plates, highlighting the primary pores as they open into the secondary pores. The third column of images shows a cross-section view of a single filter plate to highlight the radius of curvature.

**Fig. 3:**
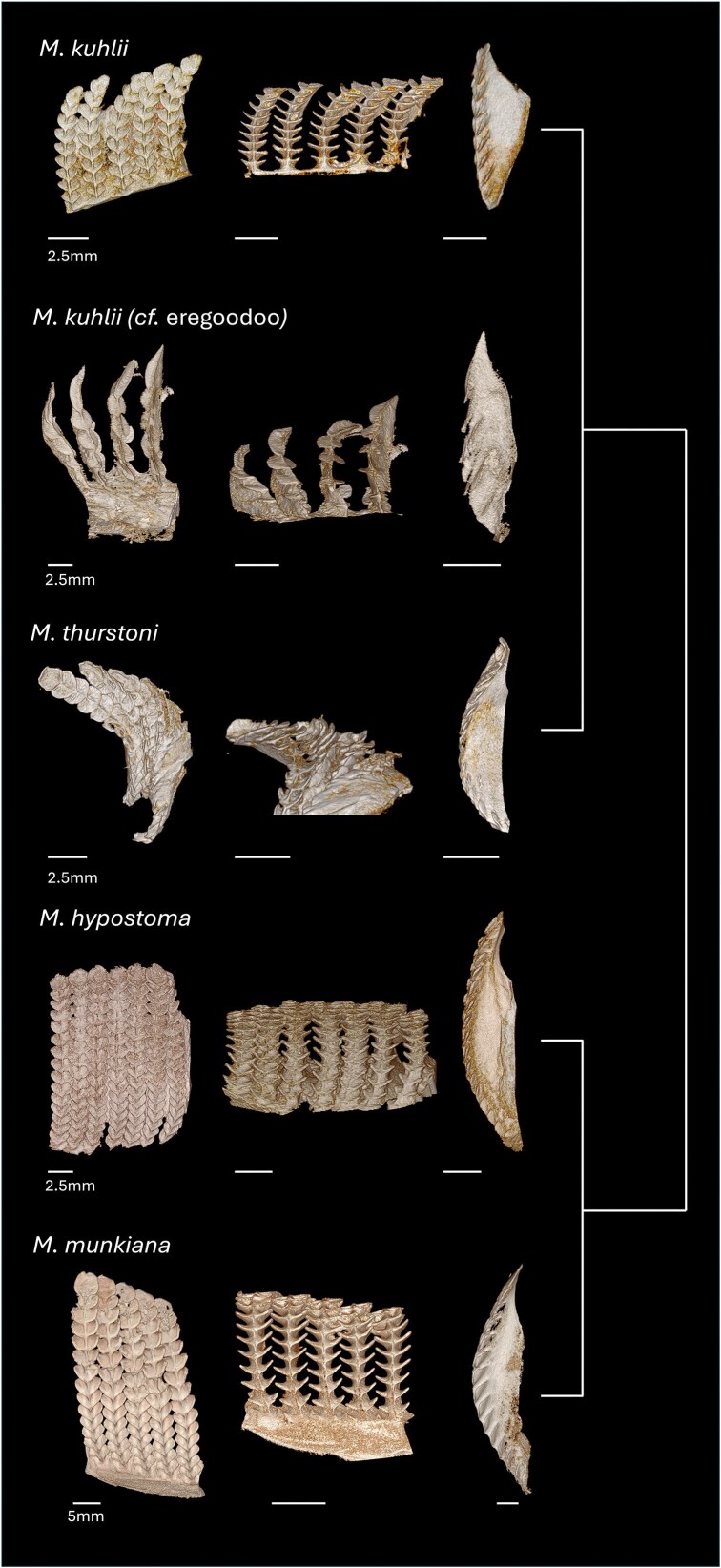
Micro-CT images of filters from five mobulid specimens in the two medium-bodied clades. *M. kuhlii* (*cf. M. eregoodoo*) and *M. thurstoni* (Poortvliet et al. 2015) in one clade, while the smallest species of mobulids, *M. hypostoma* (*cf. M. rochebrunei*) and *M. munkiana*, are in the other; phylogeny indicated on the right. The first column of images shows the posteroanterior view of the filter plates, illustrating the shape of the filter lobes along the filter plate. The second column shows the dorsoventral view of the filter plates, highlighting the primary pores as they open into the secondary pores. The third column of images shows a cross-section view of a single filter plate to highlight the radius of curvature.

*Mobula* spp. gill arch specimens were borrowed from several museum collections, including Scripps Institution of Oceanography (SIO) Marine Vertebrate Collection (La Jolla, CA, USA): *Mobula mobular* (*cf. japanica*) (previously reported as *M. japanica*; arch only), *Mobula tarapacana* (arch only), and *Mobula munkiana*; Natural History Museum, Los Angeles (Los Angeles, CA, USA): *Mobula thurstoni*; National Museum of Natural History; Smithsonian Institution (USNM) (Washington, D.C., USA): *Mobula kuhlii* (*cf. eregoodoo*) (previously reported as *M. eregoodootenke*), *Mobula kuhlii, Mobula hypostoma, Mobula mobular*, and *Mobula birostris* (previously reported as *Manta birostris*, filter lobe sample); and an additional specimen was provided by R. Rubin: *M. birostris* (arch). Specimen list available (Table 1, Figs. 2 and 3).

**Table 1:** Specimen list of individual ID, sex, disc width, arch sample size, and CT magnification (units = mm, unless otherwise indicated).

Species	Collection	Specimen ID	Sex	Disc width	Arch sample size	Magnification
*M. birostris*	R. Rubin	NA	Not specified	Not specified	268	18.8
*M. hypostoma*	USNM	205,397	Male	720	27	22.0
*M. kuhlii*	USNM	205,268	Male	779	44	23.0
*M. kuhlii (cf. eregoodoo)*	USNM	170,365	Female	722	43	28.4
*M. mobular*	USNM	372,391	Male	419*	28	18.8
*M. mobular (cf. japanica)*	SIO	54–217	Male	∼700	185	23.0
*M. munkiana*	SIO	85–34	Female	881	70	34.1
*M. tarapacana*	SIO	83–113	Female	3015	330	34.1
*M. thurstoni*	LACM	38,433–1	Female	612	37	23.0

Third branchial arch (previously removed by Paig‐Tran, Kleinteich, and Summers, 2013) from each specimen, used for re-analysis. *M. birostris, M. tarapacana*, and *M. mobular (cf. japanica)* had a section of the full arch sampled for scanning due to scanner size restraints. Macro morphology (e.g., disk length and mouth width) of specimens reported in Paig-Tran et al. 2013 and Notarbartolo-di Sciara 1987. Arch size was measured from medial to lateral-most point of the sample. *M. mobular (372,391) may be a juvenile.

It has been proposed that *M. eregoodoo* (*cf. eregoodootenkee*) and *M. kuhlii* should be synonymized, as well as *M. mobular* and *M. japanica* (Poortvliet et al. 2015; White et al. 2018), and we herein refer to samples as *M. kuhii* (*cf. eregoodoo*) (USNM 170,365) and *M. kuhlli* (USNM 205,268), and *M. mobular* (USNM 372,391) and *M. mobular* (*cf. japanica*) (SIO 54–217). However, substantial morphological differences are present between these samples, which may result from developmental differences or could indicate different morphotypes that may be inconsistent with synonymization (White et al. 2006; Cuevas-Zimbrón et al. 2013; Pardo et al. 2016; Kiyatake et al. 2025; Notarbartolo-di-Sciara 1988; Sampson et al. 2010) (Table 1).

### Micro-computed tomography

Excised filter lobes from seven *Mobula* species (9 individuals total; *M. tarapacana, M. munkiana, M. thurstoni, M. hypostoma, M. birostris, n* = 1; *M. mobular* and *M. kuhlii, n* = 2; Table 1, Figs. 2 and 3) were stained in 2.5% phosphomolybdic acid (PMA) for approximately 24 h, followed by a rinse in distilled water to remove excess stain. Once fully penetrated with the stain, samples were scanned using a Bruker Skyscan 1173 micro-CT system at the Karel F. Liem Bioimaging Center, Friday Harbor Laboratories (University of Washington). Acquired scans were reconstructed using NRecon (Micro Photonics Inc.) and segmented in 3D Slicer (Fedorov et al. 2012; V.5.8.1). All filter lobe samples were scanned at a resolution of 18–34 μm, with a source voltage of 55 kV, a source current of 160 μA, and an exposure time of 1700 ms.

Additionally, an adult *M. birostris* sample donated by Robert Rubin was scanned using an NSI X5000 system at a resolution of 102 μm (scanned at the University of Washington, Seattle, WA, USA) and reconstructed with North Star Imaging software. Full-body CT scans of *M. thurstoni, M. mobular* (*cf. M. japanica*), and *M. munkiana* were made at the UC Irvine Medical Center (Irvine, CA, USA). The specimens were scanned using a 16 slice, Siemens RS SOMATOM Sensation (MDCT-16) with 0.75 mm slice thickness and helical-spiral scans (Paig-Tran et al. 2013).

### Anatomical measurements

Filter plates are present in mobulas on the anterior and posterior sides of the ceratobranchial and epibranchial arches. In this study, we took measurements from the anterior and posterior sides of the ceratobranchial. Although measurements were collected from both sides, in some specimens the anterior ceratobranchial was too damaged to sample. Measurements collected from a posterior ceratobranchial can be found in Table 3, while data collected from an anterior ceratobranchial are available in Table 4.

**Fig. 4:**
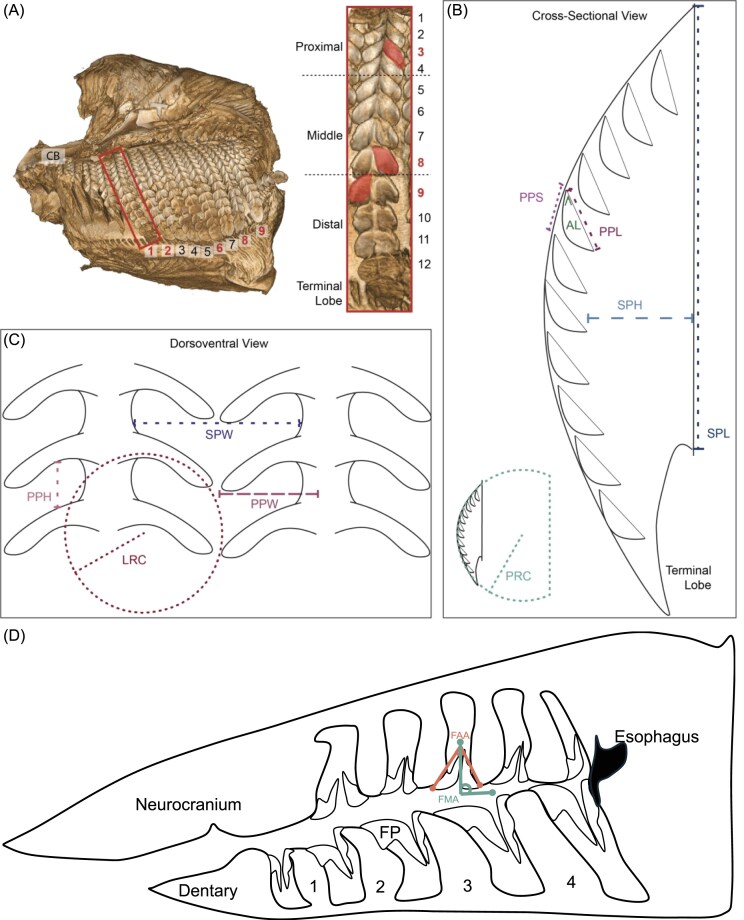
Schematic representations of methodology used for filter lobe, filter plate, and arch morphometrics. (A) Micro-CT was used to reconstruct the morphology of an excised gill arch sample [showing ceratobranchial (CB) arch from *M. munkiana*]. Five undamaged filter plates were selected for sampling. Measurements for analysis were made from filters on the posterior and anterior (when possible) side of the gill arch, and one lobe from each section of the filter plate (proximal, middle, and distal) was selected for measurements of the pore. (B) Cross-sectional view showing plate radius of curvature (PRC), primary pore period (PPP), lobe angle (LA), primary pore length (PPL), secondary pore height (SPH), and secondary pore length (SPL). (C) Focused view of posterior filter lobes showing primary pore span (PPS), lobe radius of curvature (LRC), primary pore width (PPH), secondary pore width (SPW). (D) Sagittal view of the mobula buccal cavity, from neurocranium to the esophagus, showing the filter midline angle (FMA) and the filter arch angle (FAA). The dashed line indicating FMA is referenced to the midsagittal plane and extends outward from the midline. Numbers represent the gill arches supporting the arrays of filter plates.

Filter plates were counted and numbered, starting at the most medial edge (nearest the connection to the hypobranchial) to the most lateral filter plate (nearest the connection to the epibranchial) (Fig. 4A). Five of these filter plates were arbitrarily selected from the posterior face of the ceratobranchial to measure the secondary pore dimensions. We limited our measurements to plates that showed little to no damage. We then collected morphometrics from filter lobes and filter plates that were selected using a random numbers generator. In cases where fewer than five plates were available, we sampled the maximum number available.

Following the selection of filter plates for sampling, we counted the total number of paired, ascending filter lobes on the selected filter plate. We then divided the filter plate into three equal sections designated as proximal (at the base of the filter where the central raphe originates closest to the branchial arch), middle, and distal (nearest the terminal lobe) (Fig. 4A). One lobe per section was then selected for detailed morphometric measurements using a random numbers generator. In cases where filter plate damage made it impossible to confirm lobe position, an approximation was determined using the average number of ascending lobes on neighboring plates.

The morphological aspects of the primary pore, here defined as the space between the superior surface of a filter lobe and the inferior edge of the next superiorly located filter lobe, were measured using 3D Slicer. Primary pore dimensions measured included primary pore period, width, span, and length (Fig. 4C). Primary pore span defines the space from where the lobe connects to the central raphe out to the distal most edge of the lobe. We measured the primary pore width as the distance from the tip of one lobe to the nearest point on the ascending lobe (Kahane-Rapport et al. 2025). We measured the primary pore period as the distance from the point of lobe attachment along the central raphe to the point of attachment along the central raphe in the subsequent ascending lobe. Primary pore length was measured as the total distance of attachment of the filter lobe along the central raphe.

Additional measurements of the filter lobe, the lobe radius of curvature and the lobe angle, were captured in ImageJ (v1.53k; Schneider et al. 2012; Fig. 4B; Table 3 and 4). Using a cross-sectional view of the filter plate, a reference line was drawn between the leading edge of the two lobes on either side of the target lobe (superior and inferior to the target lobe). A line was drawn through the target lobe, from the trailing edge to its leading edge. Lobe angle was measured as the angle in degrees intersecting these two lines (Fig. 4B). Additionally, we fit a curve along the leading edge of the filter lobe to estimate the lobe radius of curvature (Fig. 4B). Radius of curvature is the reciprocal of the curvature of the lobe. Finally, a circle was fitted along the line of the central raphe to estimate the plate radius of curvature. The number of measurements sampled per location is reported in Supplemental Tables S1 and S2.

We used 3D Slicer to characterize the dimensions of the secondary pore, here defined as the space between two adjacent filter plates, with a focus on the channel that is found between the central raphes of the plates (Fig. 1K). Secondary pore dimensions measured include secondary pore height, spacing, and length, and plate radius of curvature. Secondary pore height was found by measuring the distance from the base of central raphe to the inferior surface of the filter lobe at the apex of the arch. The secondary pore width was measured as the distance between two adjacent central raphes. We measured the secondary pore length as the maximum distance of the central raphe along its connection to the branchial arch (Fig. 4B, C).

We used whole body μ-CT scans of three intact mobulas [*M. munkiana, M. mobular* (*cf. japanica*), and *M. thurstoni*; Table 5] to measure gill arch angles and lengths. Ceratobranchial measurements included the hypobranchial, as we could not determine where the ceratobranchial/hypobranchial joint was located at the available resolution. We measured the ceratobranchial length as the distance from the connection to the basibranchial out to the most distal point along the arch along the joint with the epibranchial. Similar to the ceratobranchial, it was difficult to interpret the joint between the epibranchial and pharyngobranchial. Instead, we measured each epibranchial as the length from the center of the articulating surface with the ceratobranchial to the articulating surface where the epibranchial-pharyngobranchial complex connects to the chondrocranium.

We quantified the angles of the gill filter arch. To do this, we examined the individual tomographic slices and identified a standardized orthogonal plane where the anterior and posterior filter faces of each of the five paired arches were visible. This viewing plane was consistently applied across the three full-body CT scans, with the exception of the fifth gill arch in *M. thurstoni*, where this view was not accessible. For *M. thurstoni*, we identified a comparable position to the other scans for measurement consistency. We recorded two angles: the filter–arch angle, the angle between the gill arch and the anterior filter face, and the filter–midline angle, which was determined by bisecting the specimen along the midline and halving the filter–arch angle to yield the angle between the arch and the sagittal plane. All measurements taken from whole body μ-CT scans are reported as direct measured dimensions (Table 5).

Museum specimens of mobulid gill arches are exceedingly rare; for most species, we only had access to a single specimen (or two) for morphological measurements via CT scanning, limiting our ability to assess inter and intraspecific variation. However, one species, *M. hypostoma*, was available (*n* = 7) for intraspecific analysis, but not additional CT scans. Morphometrics were collected from six full-body specimens of *M. hypostoma* at the Smithsonian Museum of Natural History collection (specimen list and available measurements in Table 2). Most specimens were stored with their fins folded dorsally, as such, we measured disc width ventrally and dorsally to determine the mean disc width. Additionally, photos were taken of the filter lobes in the six full-body specimens of *M. hypostoma* and in one *M. birostris* (USNM 140,962, only the right half of the animal remains as part of the Smithsonian collection) using an endoscope (Wohler VE400 HD Video Endoscope) that was inserted into the buccal cavity through the mouth or gill slits together with a measuring scale. Due to the stiffness of the preserved specimens, the endoscope could only be inserted into each specimen to take photos of the filter lobes on the first three gill arches. The photos of *M. hypostoma* were analysed using ImageJ software to measure primary pore spanand primary pore period (max *n* = 10 for each location; 40 total measurements for each *M. hypostoma*) at different gill arches, branchials (epibranchial vs. ceratobranchial), and orientation (anterior vs. posterior). All measurements provided in Tables 6 and 7.

**Table 2:** Specimen list of *M. hypostoma* [all from the National Museum of Natural History, Smithsonian Institution (USNM)] with outer body measurements and IDs of specific gill arches (GA) where filter lobe measurements were obtained non-destructively.

*M. hypostoma* ID	Disc length (mm)	Disc width (mm)	Mouth width (mm)	Arch ID
73,871	640	1180	139	GA1, GA2
73,872	655	1198	147	GA1, GA2, GA3
73,873	623	1153.5	130	GA1
73,874	575.5	1089	125	GA2
197,409	350	673.5	82	GA1
205,397	382.5	720	85	GA1, GA2, GA3

### Statistical analyses

**Fig. 5:**
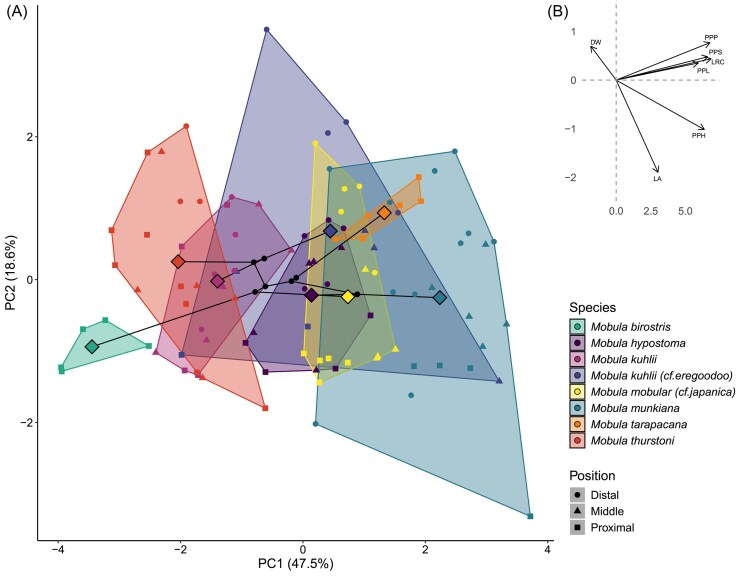
(A) A phylomorphospace visualizing the variation in primary pore morphology of eight mobula specimens (seven species). Large diamonds depict the specimen means, while smaller symbols represent pore position along the plate on the posterior side of the arch (B) Principal component loadings plot illustrating the contribution of each morphological variable to the first two principal components (PC1 = 47.5% and PC2 = 18.6%). Arrows represent the direction and magnitude of each variable's loading, indicating influence on the principal components. Variables included: disc width (DW), primary pore period (PPP), primary pore span (PPS), lobe radius of curvature (LRC), primary pore length (PPL), primary pore width (PPW), and lobe angle (LA).

**Fig. 6:**
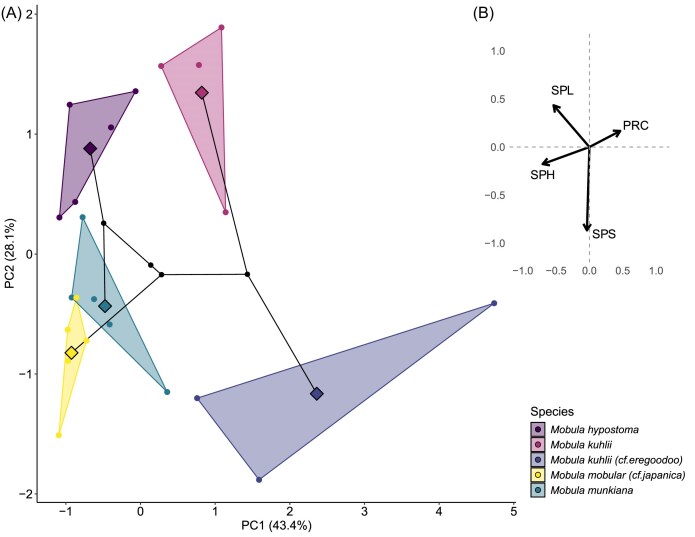
(A) A phylomorphospace visualizing the variation in secondary pore morphology of five mobula specimens (four species). Large diamonds depict the species means, while smaller symbols represent position of the pore along the plate. (B) Principal component loadings plot illustrating the contribution of each morphological variable to the first two principal components (PC1 = 43.4% and PC2 = 28.1%). Arrows represent the direction and magnitude of each variable's loading, indicating influence on the principal components. Variables included: secondary pore length (SPL), plate radius of curvature (PRC), secondary pore height (SPH), and secondary pore spacing (SPS).

We report summary statistics (mean ± SD) for *M. mobular* (*cf. M. japanica*), *M. tarapacana, M. munkiana, M. thurstoni, M. kuhlii* (*cf. M. eregoodoo*), *M. kuhlii, M. hypostoma, M. mobular*, and *M. birostris* (Tables 3, 4). To explore morphological variation across species, we performed a phylogenetically informed Principal Component Analysis (pPCA) in R (v4.3.3), focusing on primary and secondary pore dimensions (Figs. 5, 6). All morphological variables were log-transformed and scaled prior to analysis.

**Table 3:** Measurement data for posterior side of the ceratobranchial arch (mean ± standard deviation; units = mm, unless otherwise indicated).

Measurement (mm)	Position	Specimen							
		*M. birostris*	*M. hypostoma*	*M. kuhlii*	*M.kuhlii*(*cf. eregoodoo*)	*M. mobular*	*M. mobular*(*cf. japanica*)	*M. munkiana*	*M. thurstoni*
Primary pore span (PPS)	Proximal	2.00 ± 0.17	0.82 ± 0.13	0.77 ± 0.17	0.52 ± 0.13	0.45 ± 0.05	0.84 ± 0.03	1.48 ± 0.10	0.53 ± 0.10
	Middle	NA	0.92 ± 0.14	0.94 ± 0.15	0.73 ± 0.20	0.49 ± 0.04	0.97 ± 0.08	1.56 ± 0.16	0.59 ± 0.10
	Distal	NA	1.03 ± 0.04	0.97 ± 0.05	0.81 ± 0.06	0.59 ± 0.08	1.01 ± 0.04	1.63 ± 0.12	0.88 ± 0.08
Primary pore width (PPW)	Proximal	0.58 ± 0.11	0.35 ± 0.05	0.31 ± 0.06	0.75 ± 0.46	NA	0.45 ± 0.08	0.80 ± 0.10	0.28 ± 0.11
	Middle	NA	0.25 ± 0.06	0.26 ± 0.09	0.53 ± 0.28	NA	0.45 ± 0.08	0.65 ± 0.12	0.18 ± 0.05
	Distal	NA	0.31 ± 0.03	0.19 ± 0.05	0.25 ± 0.12	NA	0.27 ± 0.09	0.56 ± 0.11	0.05 ± 0.02
Primary pore period (PPP)	Proximal	1.07 ± 0.11	0.99 ± 0.07	1.07 ± 0.10	1.32 ± 0.16	0.41 ± 0.07	0.98 ± 0.06	1.52 ± 0.07	0.85 ± 0.09
	Middle	NA	1.08 ± 0.12	1.03 ± 0.15	1.56 ± 0.12	0.42 ± 0.05	1.19 ± 0.05	1.73 ± 0.13	0.89 ± 0.09
	Distal	NA	1.07 ± 0.09	0.94 ± 0.12	1.59 ± 0.15	0.40 ± 0.03	1.18 ± 0.08	1.80 ± 0.18	0.89 ± 0.11
Primary pore length (PPL)	Proximal	2.26 ± 0.15	1.28 ± 0.23	0.98 ± 0.20	1.38 ± 0.29	NA	1.27 ± 0.06	2.16 ± 0.29	1.08 ± 0.14
	Middle	NA	1.29 ± 0.04	1.07 ± 0.18	2.03 ± 0.49	NA	1.58 ± 0.22	2.31 ± 0.12	1.16 ± 0.09
	Distal	NA	1.31 ± 0.13	1.27 ± 0.14	1.90 ± 0.14	NA	1.50 ± 0.12	2.42 ± 0.17	1.14 ± 0.05
Lobe angle (LA) (deg)	Proximal	39.39 ± 2.87	37.50 ± 2.89	29.31 ± 10.89	49.36 ± 17.17	40.12 ± 2.77	38.38 ± 2.70	44.27 ± 3.23	30.65 ± 7.83
	Middle	NA	34.49 ± 7.67	29.33 ± 3.96	32.73 ± 12.35	35.83 ± 2.37	39.81 ± 4.17	41.59 ± 5.30	25.51 ± 4.73
	Distal	NA	26.21 ± 2.75	28.19 ± 3.24	18.65 ± 5.31	34.58 ± 4.22	23.45 ± 4.32	27.92 ± 6.04	25.65 ± 4.22
Lobe radius of curvature (LRC)	Proximal	2.15 ± 0.45	0.84 ± 0.26	0.58 ± 0.06	0.70 ± 0.20	0.38 ± 0.04	0.99 ± 0.19	1.06 ± 0.10	0.34 ± 0.07
	Middle	NA	0.94 ± 0.15	0.75 ± 0.19	0.80 ± 0.28	0.36 ± 0.02	0.94 ± 0.208	1.36 ± 0.17	0.37 ± 0.10
	Distal	NA	0.97 ± 0.09	0.72 ± 0.14	0.85 ± 0.07	0.61 ± 0.17	1.14 ± 0.16	1.28 ± 0.20	0.68 ± 0.16
Secondary pore width (SPW)	Proximal	NA	0.71 ± 0.21	0.64 ± 0.18	1.27 ± 0.49	NA	1.48 ± 0.16	1.63 ± 0.16	NA
	Middle	NA	1.18 ± 0.25	0.98 ± 0.09	1.86 ± 0.20	NA	1.53 ± 0.25	2.56 ± 0.34	NA
	Distal	NA	1.31 ± 0.25	1.43 ± 0.05	2.21 ± 0.27	NA	1.70 ± 0.25	2.31 ± 0.26	NA
Secondary pore height (SPH)	–	NA	2.57 ± 0.27	2.07 ± 0.38	1.19 ± 0.59	NA	3.51 ± 0.29	4.36 ± 0.53	NA
Secondary pore length (SPL)	–	NA	11.77 ± 0.92	11.62 ± 1.40	8.17 ± 0.42	NA	9.59 ± 0.11	16.62 ± 1.09	NA
Plate radius of curvature (PRC)	–	NA	10.72 ± 0.72	11.70 ± 2.08	8.87 ± 2.15	NA	9.08 ± 0.37	16.06 ± 0.71	NA

*Mobula birostris* only has proximal primary pore data and limited secondary pore data because only a small section of the arch could be micro-CT scanned. *M. mobular* had fused lobes making some measurements unobtainable; damaged plates made secondary pore data for *M. thurstoni* unobtainable. Posterior side of *M. tarapacana* was not sampled from. Note that deg refers to degree, n.d. refers to no dimensions.

**Table 4:** Measurement data for the anterior side of the ceratobranchial arch (mean ± standard deviation; units = mm, unless otherwise indicated).

Measurement (mm)	Position	Specimen				
		*M. mobular*	*M. mobular* (*cf. japanica*)	*M. munkiana*	*M. tarapacana*	*M. thurstoni*
Primary pore span (PPS)	Proximal	NA	0.86	NA	3.55 ± 0.43	0.58 ± 0.10
	Middle	0.52 ± 0.03	0.95	NA	NA	0.57
	Distal	0.53 ± 0.001	0.78	1.29 ± 0.13	NA	0.60 ± 0.08
Primary pore width (PPW)	Proximal	NA	0.52	NA	1.06 ± 0.29	0.15 ± 0.07
	Middle	NA	0.41	NA	NA	0.34
	Distal	NA	0.31	0.61 ± 0.11	NA	0.10 ± 0.03
Primary pore period (PPP)	Proximal	NA	0.939	NA	2.97 ± 0.57	0.80 ± 0.10
	Middle	0.41 ± 0.02	0.80	NA	NA	0.86
	Distal	0.42 ± 0.02	1.04	1.40 ± 0.15	NA	0.85 ± 0.03
Primary pore length (PPL)	Proximal	NA	NA	NA	4.48 ± 0.56	1.07 ± 0.08
	Middle	NA	NA	NA	NA	0.99
	Distal	NA	NA	1.74 ± 0.22	NA	1.00 ± 0.14
Lobe angle (LA) (deg)	Proximal	NA	38.87	NA	34.64 ± 2.15	20.65 ± 1.94
	Middle	33.82 ± 4.23	45.91	NA	NA	34.27
	Distal	32.36	32.76	34.60 ± 12.96	NA	17.25 ± 3.22
Lobe radius of curvature (LRC)	Proximal	NA	0.75	NA	3.98 ± 1.20	0.40 ± 0.10
	Middle	0.48 ± 0.07	0.85	NA	NA	0.38
	Distal	0.39 ± 0.06	0.59	1.12 ± 0.32	NA	0.44 ± 0.08
Secondary pore width (SPW)	Proximal	NA	1.23	NA	4.67 ± 0.66	NA
	Middle	NA	0.95	2.01 ± 0.06	NA	NA
	Distal	NA	NA	2.23 ± 0.01	NA	NA
Secondary pore height (SPH)	–	NA	1.39	1.84 ± 0.12	6.46 ± 1.10	NA

*Mobula tarapacana* only has proximal primary pore data and limited secondary pore data because only a small section of the arch was micro-CT scanned. *Mobula mobular* had fused lobes making some measurements unobtainable; damaged plates made secondary pore data for *M. thurstoni* unobtainable. Anterior side of *M. birostris, M. hypotsoma*, and *M. kuhlii* were unable to be sampled from. Plate radius of curvature (PRC) was only obtained from posterior plates. Number of measurements collected in Supplemental Table S2. Note that deg refers to degree, n.d. refers to no dimensions.

To test for differences due to position on a filter plate (proximal, middle, or distal), we performed repeated-measures ANOVAs for each primary pore measurement in R. Position was treated as a within-subject factor to account for the non-independence of the measurements. When a significant effect due to position was detected, a pairwise post-hoc comparison was conducted using estimated marginal means with Bonferroni correction for multiple comparisons.

**Fig. 7:**
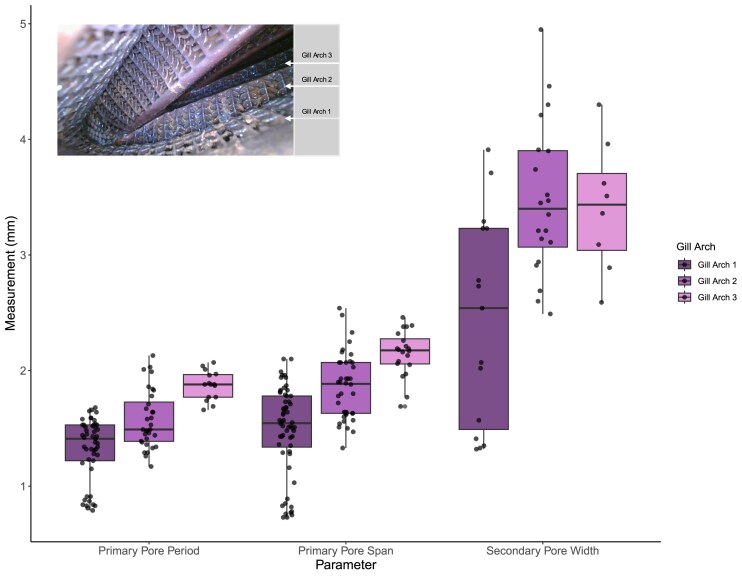
Filter lobe and plate measurements from six *M. hypostoma* specimens based on photos taken with an endoscope. Individual measurements of primary pore period, primary pore span, and primary pore width (reported in mm) for Gill Arches 1, 2, and 3, shown from left to right. Measurements of primary pore period (dark purple), primary pore span (medium purple) and secondary pore width (light purple) reported in mm. Each point represents an individual measurement on a gill arch (1–3). Inset photo from a *M. hypostoma* specimen (73,871), captured by endoscope, indicating the arrangement of gill arches.

As *M. hypostoma* had seven specimens available, we reported summary statistics (mean ± SD) (Table 6) and ran linear mixed models to compare primary and secondary pore measurements with disc width, branchial arch position, and gill arch number (Table 7, Fig. 7). Disc width (mm) was centered on its mean to facilitate interpretation of model parameters and reduce potential multicollinearity. Models were fit by restricted maximum likelihood using the lmer function from the “lme4” package in R, with *P*-values for fixed effects estimated via Satterthwaite's method. We compared candidate models using Akaike Information Criterion (AIC) to select the most parsimonious model. We also conducted intraclass correlation coefficient (ICC) tests in R using the “performance” package to clarify how much variation can be explained by the random effects and where the variation lies. We ran linear mixed-effects models (LMMs) to test whether the measured variable (primary pore span, primary pore period, or secondary pore width) significantly differed across the gill arches, while adjusting for disc width and accounting for the nested structure of repeated measures within individuals and branchial units. The nested random effects structure captured variability both at the individual level and across branchial arches within *M. hypostoma* individuals (Fig. 7).

## Results

### Interspecies variation in primary pore morphology

**Fig. 8:**
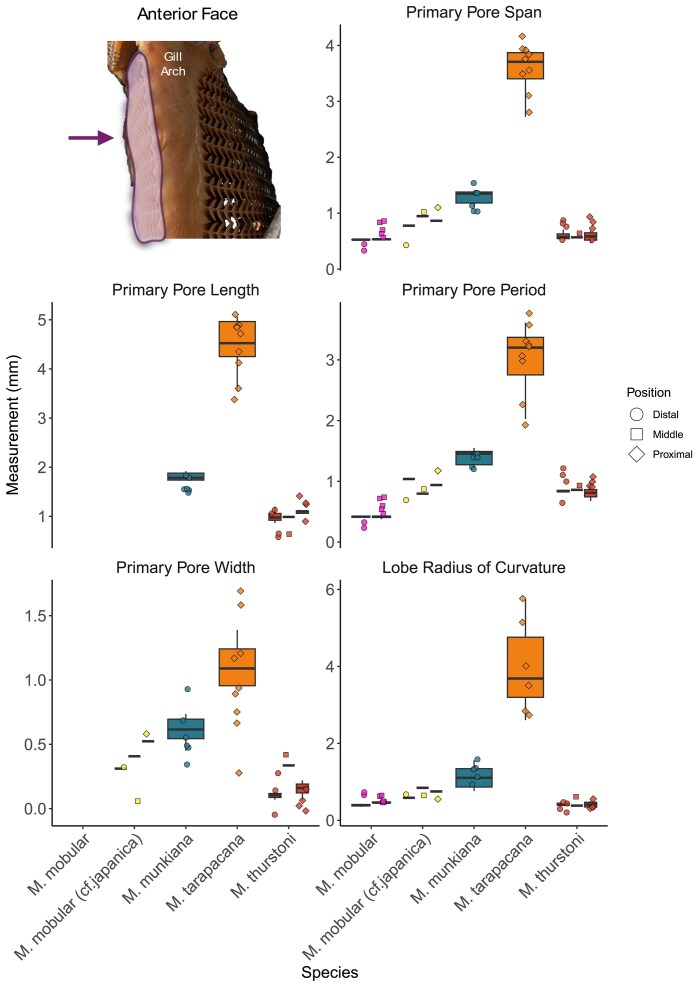
Filter lobe morphometrics measured from five specimens on the anterior face of the gill arch (inset photo). Boxplots show the distribution of the data, the central line representing the median, while the edges of the box represent the first and third quartiles. Each point represents a filter lobe haphazard selected for measurement, symbol shapes indicate the position along the plate. Individual specimens are listed left to right as; *M. mobular, M. mobular (cf. japanica), M. munkiana, M. tarapacana*, and *M. thurstoni*.

**Fig. 9:**
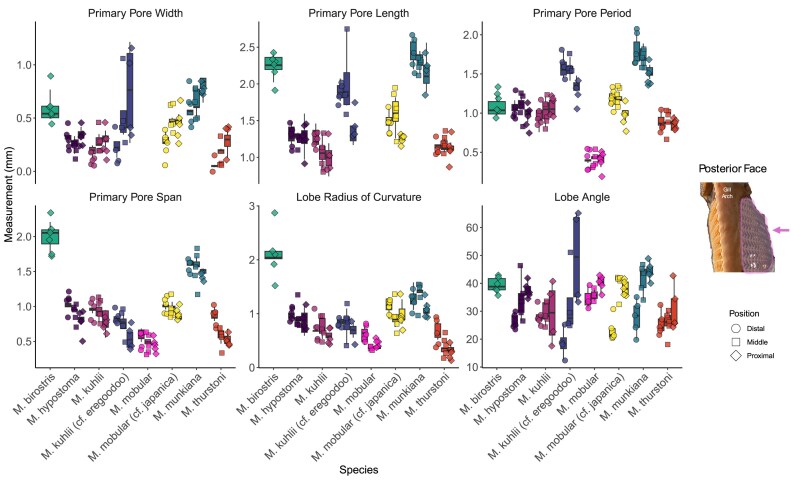
Filter lobe morphometrics measured from eight specimens on the posterior face of the gill arch (inset photo). Boxplots show the distribution of the data, the central line representing the median, while the edges of the box represent the first and third quartiles. Each point represents a filter lobe haphazard selected for measurement, symbol shapes indicate the position along the plate. Individual specimens are presented left to right as; *M. birostris, M. hypostoma, M. kuhlii , M. kuhlii* (*cf. eregoodoo*), *M. mobular, M. mobular* (*cf. japanica*), *M. munkiana, M. thurstoni*.

Micro-CT images of museum specimens characterized the morphological variation of the primary pore across seven mobulid species (eight total specimens). Consistent with prior qualitative surveys, we found variation in the size and shape of the filter lobes across the species (Figs. 8, 9; Paig-Tran 2013). The dimensions of the posterior ceratobranchial primary pores were relatively conserved across species (Table 3, Fig. 9). Measurements of primary pore width and lobe angle showed a trend of decreasing from proximal to distal, while measurements of primary pore length, period, span and lobe radius of curvature showed a trend of decreasing from distal to proximal. However, the only significant differences in primary pore dimensions found were between the proximal and middle radii of curvature in *M. kuhlii* (*P = 0.0162*), between the proximal and distal pore spans (*P* = 0.0491) and the middle and distal radii of curvature (*P = 0.0277*) in *M. mobular*, between the proximal and distal lobe periods (*P = 0.0328*) in *M. munikana*, and between the proximal and distal lobe spans (*P = 0.0496*) in *M. thurstoni*. More sampling is necessary to determine whether this is a conserved feature across the species or unique to these specimens (Supplemental Table S1). The dimensions of the primary pore of the anterior ceratobranchial lobes exhibited some variation in the proximal to terminal direction, with linear dimensions tending to increase toward the terminal edge of the filter plate (Fig. 8). Due to the lack of measurable lobes on the anterior filter array, this trend could not be validated statistically (Supplemental Table S2, Fig. 8).

The primary pore width exhibited the greatest variation across species (Fig. 9). The smallest mean pore width was 0.05 ± 0.02 mm observed in the distal lobes of *M. thurstoni*, and the largest pore width was 0.80 ± 0.10 mm in the proximal lobes of *M. munkiana*. Primary pore period exhibited the least amount of variation. The smallest pore period (∼0.41 ± 0.07 mm) was measured in *M. mobular* proximal pores and the largest pore period (1.80 ± 0.18 mm) was recorded in *M. munkiana* distal pores (Table 3).

To better understand the relationships between all the primary pore dimensions, we used phylogenetically corrected PCA analysis to examine the primary pore morphospace (Fig. 5). The first principal component (PC1) explains 47.5% of the total variance, while the second component (PC2) accounts for an additional 18.6%. The third component (PC3) contributes an additional 13.8%, resulting in 79.9% of the total variance explained by the first three components. PC1 primarily captures pore dimension variation between the examined specimens. PC1 loading is almost uniformly distributed across the linear dimensions, including lobe radius of curvature (loading = 0.457), primary pore period (0.452), primary pore span (0.441), primary pore width (0.425), and primary pore length (0.396). This suggests that PC1 reflects differences in the overall pore size between specimens, where higher PC1 scores correspond to specimens with larger and more widely spaced primary pores. PC2 tends to capture morphometric variation along the proximal-terminal axis. PC2 loading values indicate changes in the pore shape, particularly the angle of the lobe (loading −0.758), primary pore width (−0.407), and primary pore period (0.307). Filter pores with lower PC2 scores tend to have higher lobe angles and reduced pore width, whereas higher PC2 scores are associated with increased pore period. PC3 is primarily dominated by disc width (loading 0.911). This component appears to reflect variation in body shape, specifically differences in disc width, with the angle of the lobe contributing to a lesser extent (loading 0.249).

Overall, these results suggest substantial primary pore and filter lobe variation across species. PC1 reflects increase in pore length, period, span, and radius of curvature, indicating that species differ in filter size and area. PC2 captures variation in lobe angle and pore width, suggesting further shape differences among species in the orientation and vertical extent of their filtering structures. Although body size is a likely covariate influencing filter lobe morphology, the aim of this analysis was to capture the full range of morphological variation, rather than isolating shape alone, so we did not size-correct the data. This allows us to assess whether observed differences in primary pore dimensions reflect meaningful biological divergence rather than simply scaled versions of the same form. Importantly, disc width loads strongly on a distinct PC3, indicating that size-related variation is partitioned within the data and can be interpreted separately from other aspects of filter lobe morphology.

### Interspecies variation in secondary pore morphology

**Fig. 10:**
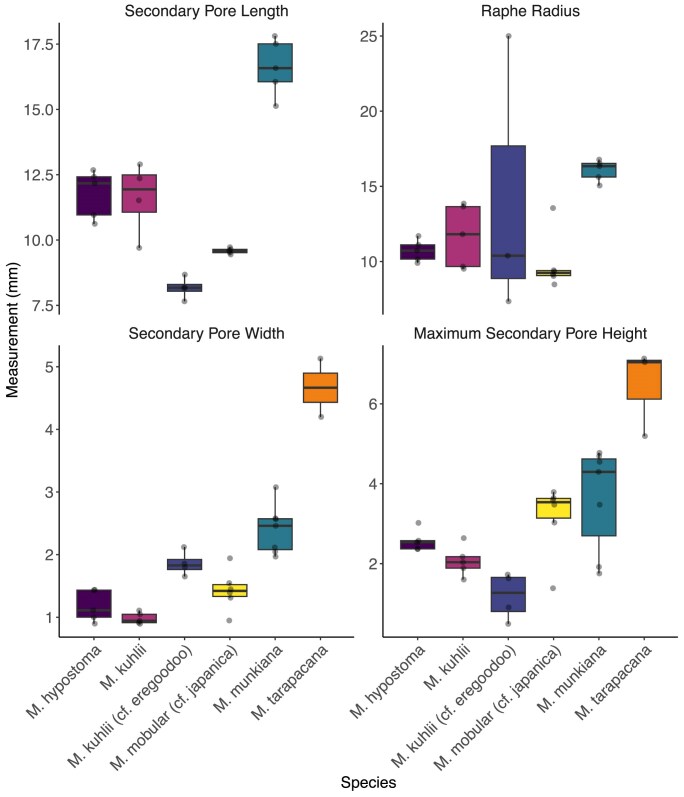
Secondary pore morphometrics the anterior side of the gill arch from five mobulid specimens. Each point represents a filter plate randomly selected for measurement. Individual data in each secondary pore measurement category for each species are presented from left to right; *M. hypostoma, M. kuhlii, M. kuhlii* (*cf. eregoodoo*), *M. mobular* (*cf. japanica*), *M. munkiana*, and *M. tarapacana*.

We used micro-CT to examine the morphology of the secondary pore. This analysis was performed on the five examined specimens with intact secondary pores. Since the secondary pore spans the proximal–distal axis, the data were not separated into proximal–distal sections. Similar to the primary pore, we found inter-species variation in the secondary pore dimensions (Fig. 10). The largest secondary pore height was 6.46 ± 1.10 mm in *M. tarapacana* (Table 4) and the smallest secondary pore height was 1.19 ± 0.59 mm in *M. kuhlii* (*cf. eregoodoo*) (Table 3). The longest secondary pore length was 16.62 ± 1.09 mm in *M. munkiana* (Table 3) and the shortest pore length was 8.17 ± 0.42 mm in *M. kuhlii* (*cf. eregoodoo*) (Table 3).

To further understand the secondary pore morphospace, we analyzed the pore dimensions using phylogenetically corrected PCA (Fig. 6). The first principal component (PC1) explains 43.4% of the total variance, separating species by a combination of plate radius of curvature (positive) and secondary pore height and length (negative). PC2, which explains 28.1% of the variance, mostly separates based on secondary pore spacing (strong negative influence) and secondary pore length. For PC1, the most important variables are secondary pore height (loading −0.70), secondary pore length (loading −0.54), and plate radius of curvature (loading 0.46). Species with high plate radius of curvature and smaller secondary pore height and length score higher on PC1, which may represent a size/shape trade-off. For PC2, secondary pore spacing (loading −0.87) dominates with a strong negative influence, while secondary pore length (loading 0.43) has a moderate influence. PC2 reflects pore spacing, separating species based on how far apart their secondary pores are.

### Intraspecific variation in primary and secondary pore dimensions in M. hypostoma

To address intraspecific variation, we identified a species for which multiple museum specimens were available, *M. hypostoma* (Table 2). Using LMMs, we found that primary pore span increases progressively along the gill arches, with the widest pores located on the third gill arch (gill arch 2: β = 0.295 mm, *P* < 0.001; gill arch 3: β = 0.695 mm, *P* < 0.001). We then assessed variation in primary pore period across different gill arches. Primary pore period was significantly influenced by gill arch position. Compared to the first gill arch, period was significantly greater on both the second (β = 0.249, *P* < 0.001) and third gill arches (β = 0.631, *P* < 0.001). We analyzed the variation in secondary pore width across gill arches. The model revealed that gill arch number has a significant effect on secondary pore width (*P* < 0.001). On average, secondary pores located on gill arch 2 were 1.39 mm wider than those on gill arch 1 (β = 1.39, *P* < 0.001). Similarly, secondary pores on gill arch 3 were 1.35 mm wider than those on gill arch 1 (β = 1.35, *P* < 0.001). Across all three models, disc width had a small, marginally positive effect.

To further test for intraspecific variation, we conducted an ICC test. Our ICC indicated that ∼60% of variance in the models is due to differences between gill arches within an individual. This suggests that the traits, primary pore span, primary pore period, and secondary pore width, exhibit significant variation across different gill arches of the same individual but are very conserved across *M. hypostoma*.

### Variation in filter morphology along anterior–posterior axis

Across all specimens, the posterior filter plates are strongly convex in their curvature with an almost shelf-like appearance along the proximal portion (Fig. 1E–G) (average plate radius of curvature was 11.62 ± 2.90 mm). Direct measurements showed that *M. munkiana* filter plates displayed the greatest plate radius of curvature (16.06 ± 0.71 mm; Table 3) and *M. kuhlii* (*cf. eregoodoo*) and *M. mobular* (*cf. japanica*) had the smallest plate radius of curvatures (∼9 mm; Table 3). Anterior filter plates were straighter compared to posterior plates and no radius of curvature was obtained (measured) from them. Due to this asymmetry, the posterior filter plate and anterior filter plate of the adjacent gill arch have the potential to form deep channels or passages through which water flows during filter feeding (Fig. 1E, F).

**Fig. 11:**
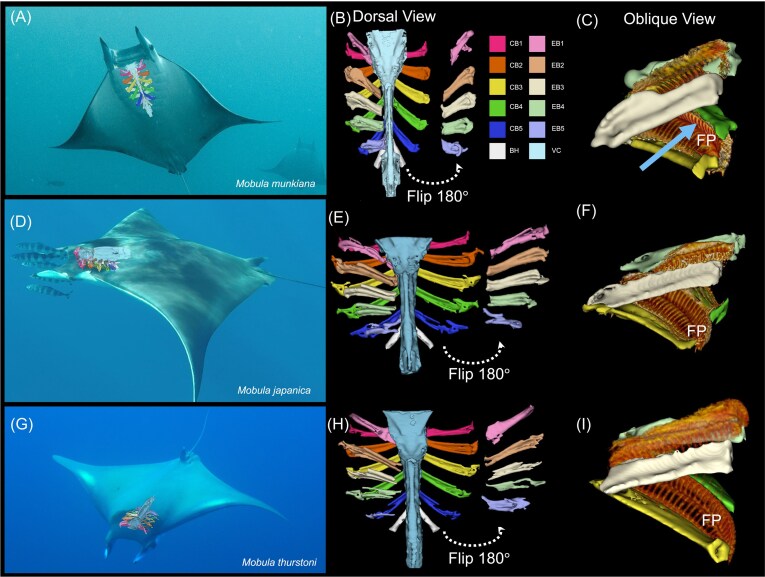
Comparative skeletal structures of three *Mobula* species. (A, D, G) Photographs of *Mobula munkiana* (credit: Simon Pierce, https://naturetripper.com), *Mobula mobular* (formerly *M. japanica*; credit: Clinton Duffy), and *Mobula thurstoni* (credit: iNaturalist), respectively, with overlaid visualizations of their cranial and branchial skeletal elements. (B, E, H) Dorsal views of the cranial and branchial skeletal elements of the corresponding species, with individual bones color-coded for clarity. Anterior (rostral) toward the top, posterior (caudal) toward the bottom. In each dorsal view, the right-side epibranchial elements have been removed and flipped 180° to expose the dorsal surface of the ceratobranchials and the ventral surface of the epibranchials. Dashed arrows indicate the direction of skeletal component rotation. CB = ceratobranchial elements; EB = epibranchial elements; BH = basihyal; VC = dorsal element of the visceral cranium. (C, F, I) shows epibranchials and ceratobranchials with filter plates (FP). Solid arrow in (C) represents the presumed direction of flow over the plates for all species.

To better visualize these channels, CT images were constructed for the complete branchial basket of three mobulid species including *M. munkiana, M. mobular* (*cf . japanica*), and *M. thurstoni* (Fig. 11). All three species exhibited pronounced anterior–posterior asymmetry in the same orientation of the gill arches. In a non-feeding position, when the mouth is closed and the buccal cavity is collapsed, the curvatures of adjacent gill arches fit together leaving a narrow channel between the filter lobes that lead toward the parabranchial chambers (Fig. 11). In addition, there were notable overall shape changes in arch anatomy across *M. munkiana, M. mobula* (*cf . japanica*), and *M. thurstoni*. In particular, *M. munkiana* had a narrower profile in dorsal view compared to the other two mobulids (Fig. 11). Comparative measurements of ceratobranchial and epibranchial elements and associated angles were obtained from each of the five branchial arches for each specimen (Table 5 ). In *M. mobular*, ceratobranchial filter-arch angles ranged from 36.1° to 56.9°. *Mobula thurstoni* showed a slightly smaller range of 30.6° to 45.4°, while *M. munkiana* presented the smallest angles, ranging from 19.2° to 36.2°. As with ceratobranchial filter-arch angles, *M. mobular* had the largest epibranchial filter-arch angles, while *M. munkiana* exhibited the smallest angles (Table 5). The epibranchial filter-midline angles in *M. munkiana* displayed both the lowest and highest angles recorded.

**Table 5:** Measurements of left branchial arches collected from full-body CT scans of *M. thurstoni, M. mobular* (*cf. japanica*), and *M. munkiana* (Paig-Tran et al. 2013).

Specimen	Arch ID	Measurements						
		Mouth width (mm)	Ceratobranchial (CB) (mm)	Epibranchial (EB) (mm)	CB filter-arch angle	EB filter-arch angle	CB filter-midline angle	EB filter-midline angle
*M. mobular (cf. japanica)*	1	103.9	63.81	66.92	39.9°	48.3°	96.4°	42.3°
	2	103.9	62.89	71.31	53.4°	44.3°	102.8°	87.0°
	3	103.9	66.85	64.96	56.9°	41.8°	107.6°	99.7°
	4	103.9	62.97	50.49	39.0°	63.0°	101.9°	109.0°
	5	103.9	50.94	45.18	36.1°	33.5°	92.2°	93.8°
*M. thurstoni*	1	110.08	56.53	72.72	43.2°	41.5°	97.4°	96.8°
	2	110.08	66.09	76.3	45.4°	48.9°	103.9°	117.1°
	3	110.08	68.3	73.05	32.8°	44.4°	114.8°	109.9°
	4	110.08	64.44	72.67	30.6°	36.5°	111.1°	116.1°
	5	110.08	48.15	52.63	38.6°	31.3°	102.5°	95.2°
*M. munkiana*	1	122.5	63.41	79.1	36.2°	39.6°	100.8°	88.0°
	2	122.5	78.49	92.77	25.9°	26.5°	118.2°	103.8°
	3	122.5	89.82	84.61	35.2	37.4°	113.8°	75.8°
	4	122.5	69.02	68.59	26	42.1	102.4°	76.8°
	5	122.5	83.65	44.68	19.2	19.7	101.8°	52.4

**Table 6:** Measurement data for *M. hypostoma* (*n* = 6; mean ± standard deviation; units = mm, unless otherwise indicated).

*M. hypostoma*	Arch ID	Location	Proximal pore measurement (mm)		
			Primary pore period	Primary pore span	Secondary pore width
73,871	1	Anterior Ceratobranchial	1.40 ± 0.17	1.54 ± 0.10	2.81 ± 0.43
73,871	2	Anterior Ceratobranchial	1.89 ± 0.20	2.20 ± 0.20	3.88 ± 0.44
73,872	1	Anterior Ceratobranchial	1.35 ± 0.09	1.79 ± 0.16	2.62 ± 0.34
73,872	1	Anterior Epibranchial	1.51 ± 0.15	1.45 ± 0.10	2.43 ± 0.36
73,872	2	Anterior Ceratobranchial	1.40 ± 0.11	1.74 ± 0.16	3.88 ± 0.78
73,872	3	Anterior Ceratobranchial	1.92 ± 0.10	2.25 ± 0.14	3.51 ± 0.68
73,873	1	Anterior Epibranchial	1.54 ± 0.07	1.82 ± 0.30	3.47 ± 0.32
73,874	2	Anterior Ceratobranchial	1.73 ± 0.21	1.92 ± 0.16	3.26 ± 0.48
197,409	1	Anterior Ceratobranchial	1.45 ± 0.14	1.53 ± 0.22	2.67 ± 0.36
205,397	1	Anterior Epibranchial	0.85 ± 0.04	0.79 ± 0.05	1.40 ± 0.10
205,397	2	Anterior Ceratobranchial	1.46 ± 0.16	1.57 ± 0.14	2.89 ± 0.34
205,397	3	Anterior Ceratobranchial	1.77 ± 0.10	2.05 ± 0.21	3.25 ± 0.32
74,280	1	Posterior Ceratobranchial	1.56 ± 0.18	1.87 ± 0.16	3.17 ± 0.45
74,280	1	Posterior Epibranchial	1.33 ± 0.08	1.41 ± 0.11	2.57 ± 0.13
74,280	2	Posterior Ceratobranchial	1.80 ± 0.10	1.86 ± 0.19	2.73 ± 0.17

Both anterior and posterior side data, from either the epibranchial or ceratobranchial arch, are included.

**Table 7:** Linear mixed-effects model results predicting primary pore span, primary pore period, and secondary pore width as a function of gill arch position and disc length. P-values less than 0.05 are shown in bold.

Parameter	Predictor	Estimate	CI	*P*	Conditional R^2^
Primary pore span (PPS)	Gill Arch 1 (Intercept)	1.52	1.32–1.71	**<0.001**	0.794
	Disc Width (Centered)	0.00	−0.00–0.00	0.050	
	Gill Arch 2	0.29	0.18–0.41	**<0.001**	
	Gill Arch 3	0.69	0.55–0.84	**<0.001**	
Primary pore period (PPP)	Gill Arch 1 (Intercept)	1.37	1.22–1.53	**<0.001**	0.835
	Disc Width (Centered)	0.00	−0.00–0.00	0.107	
	Gill Arch 2	0.25	0.16–0.34	**<0.001**	
	Gill Arch 3	0.63	0.52–0.74	**<0.001**	
Secondary pore width (PPW)	Gill Arch 1 (Intercept)	2.27	1.63–2.90	**<0.001**	0.823
	Disc Width (Centered)	0.00	−0.00–0.01	0.139	
	Gill Arch 2	1.39	0.96–1.81	**<0.001**	
	Gill Arch 3	1.35	0.89–1.81	**<0.001**	

Random intercepts were included for individual and branchial structure nested within individual. Estimates, confidence intervals, *P*-values, and conditional R^2^ are reported.

Endoscopic examination of preserved *M. hypostoma* and *M. birostris* confirmed that the posterior side of the arch in these species was also strongly curved, with the proximal edge being nearly parallel to the anterior–posterior axis while the distal edge was nearly parallel to the dorso-ventral axis (Fig. 1F). The anterior side of the arch was more concave and appeared to be nearly parallel to the dorso–ventral axis over its full surface. The face of the filter is in the same plane as the branchial arch and due to the curvature of the entire plate, the filter appears anterior facing at the most proximal point.

## Discussion

This study builds on earlier morphological descriptions of mobulid filters by creating new detailed anatomical descriptions to map the morphology of the filter plates, individual filter lobes, and pore dimensions (Bigelow and Schroeder 1953; Notarbartolo-Di-Sciara 1987; Paig‐Tran et al. 2013; Paig‐Tran and Summers 2014). Through comparisons across seven species, we found variation in primary pore period, primary pore span, and primary pore width, but the overall form is largely conserved (Figs. 8, 9). Our results indicate that the branchial filters in mobulids are highly variable across hierarchical levels: there is morphological variation across species, within the same species, variation across the branchial arches in a single individual, and variation in shape along a singular filter plate. In *M. hypostoma*, we found clear differences in the pore dimensions across the gill arches, with primary pore span, primary pore period, and secondary pore width measurements increasing in size further into the buccopharynx (Fig. 7 ). This suggests that comparative studies between species must carefully account for the gill arch and position of the filter lobes being measured.

Comparing the measurements in this study to the results from earlier simplified models (Divi et al. 2018; Kahane-Rapport et al. 2025) provides insights into how filtration performance may vary between mobulid species. Pore spacing from this study remained relatively similar across the mobulids at approximately 1160 ± 620 µm. Prior studies have used CFDs models and flow tank experiments to examine pore sizes > 1000 µm, and found that the size selectivity of the filter was not strongly dependent on pore width and the particle cutoff diameter was in the range of 250–600 µm depending on flow conditions (Kahane-Rapport et al. 2025). Based on these results, we predict that species with larger pore widths (>1000 µm) will target zooplankton and/or ichthyoplankton at or above ∼600 µm with nearly 100% efficiency (Paig-Tran et al. 2011; Kahane-Rapport et al. 2025). Several of the mobula species we examined had pore widths smaller than those examined in prior CFD and physical modeling studies (Table 3). Further studies are needed to understand the fluid dynamics of filtration in these species, but it is possible that these species target smaller plankton. The average lobe angles remained consistent across the mobulids at ∼33°±10° (averages across species ranged from 25–39°). Prior studies have examined the flow fields and filtration efficiency of *M. tarapacana* filters with varied lobe angles (27–57°) (Divi et al. 2018). In the wing-like orientation, decreased lobe angles resulted in captive vortices forming deeper in the primary pore while greater lobe angles resulted in shallower captive vortices (Kahane-Rapport et al. 2025). Lobe angles near 40° enabled filtration of smaller particle sizes (≥0.5 mm), while decreased lobe angles near 27° effectively filtered only larger particles (≥1.1mm). The flow patterns were similar in both the spoiler and wing orientation; however, the overall filtration efficiency of smaller particles (∼0.3 mm) was increased in the spoiler orientation. The measured lobe angles from this study reinforce our predictions that mobulids are likely targeting large zooplanktons and/or ichthyoplankton, but differences in the filter lobe morphology may suggest some dietary specialization. We are only beginning to understand the full relationship between form and function in mobulid filter feeding, and any predictions must be made cautiously. However, based on our present understanding, our results could indicate that *M. kuhlii* (average lobe angle ∼30°) and *M. thurstoni* (average lobe angle ∼27°) would potentially be limited to feeding on larger prey (>1 mm) while *M. birostris* (average lobe angle of 39°), *M. mobular* (average lobe angle ∼37°), and *M. munkina* (average lobe angle ∼38°) may be capable of targeting smaller sizes (as small as 0.5 mm). Such considerations would only indicate the smallest plankton that may be targeted, though mobulid fishes would certainly also be capable of feeding on larger plankton. Indeed, *M. munkiana* have been documented targeting mysid shrimps an order of magnitude larger than the lowest size-ranges they appear capable of filtering (*Mysidium* spp.; 5–25 mm; Porsiel et al. 2021). Gut content analysis has also indicated that *M. mobular* (*cf. japanica*) consume tropical krill (*P. latifrons*; 8–10 mm) and *Mobula thurstoni* feed on euphausiid shrimps (*N. simplex*; 8–16 mm) during summer and on mysids during winter (Notarbartolo-di-Sciara 1988; Couturier et al. 2012; Masangcay et al. 2018). However, we note that dietary studies are relatively rare and it is entirely plausible that mobulids target larger-sized plankton when plentiful, but use their filters to capture smaller sized particles when filtering outside of a plankton bloom. Overall, our results indicate that the pore dimensions can provide important insights into the functional properties of the filtering apparatus, and that models based on these dimensions may be useful for predicting the ability of different mobulid species to consume varying plankton types in the absence of gut contents or isotopic data.

It is still unclear exactly how water moves through the buccal cavity. We predict that the shape and orientation of the filter plates and arches have important ramifications for our understanding of the buccal flow patterns. Species like *M. mobular*, with larger ceratobranchial and epibranchial branchial arch angles, likely exhibit broader separation between filter surfaces, which may facilitate higher volumetric flow, compared to the narrower branchial arch angles in *M. munkiana* which likely constrain the inter-lobe space.

In prior studies, it was speculated that the filter surfaces had a more symmetric and flattened orientation, such that water flowing into the buccal cavity moved tangentially to the filter surfaces. Based on this understanding, it was hypothesized that fluid encountered the anterior filter lobes in a spoiler-like orientation (pore aligned with freestream flow) and the posterior filter lobes in a wing-like orientation (pore faces into freestream flow). However, our observations of the pronounced asymmetry of the gill arches, interspecific variation in branchial arch morphology and filter lobe orientation, and their arrangement within the buccal cavity point to alternative flow dynamics. The first is that the convex and concave pairing of the filter faces support unidirectional flow between the anterior and posterior filters of adjacent gill arches (Fig. 1F). Under such conditions, we would anticipate that both the anterior and posterior filter lobes would have a wing-like orientation relative to the local freestream flow. The large radius of curvature of the posterior filter plates likely reduces flow separation, allowing a more laminar flow between the two filter surfaces. A second possibility is that when the arches are shifted into the widened feeding position, the space between the filtering faces becomes enlarged. This larger space may allow the formation of large captive vortices in the space between adjacent gill arches. In this scenario, the posterior filter plates would be expected to encounter the freestream flow in the spoiler orientation, while the anterior facing plates would operate in a wing-like configuration.

Morphometric studies powered by CT imaging provide an important foundation for understanding form-function relationships and fueling the biomimetic design process (Broeckhoven and du Plessis 2022). The morphologies described in this study are expected to enable the development of more detailed 3D computational and physical models, which will likely reveal new insights into how morphological features affect filtration dynamics. In particular, it would be interesting to examine the functional implications of the gill arch variation found in *M. hypostoma*, the surface textures observed in *M. tarapacana* (Paig-Tran et al. 2013), or the finger-like projections found in *M. birostris* (Paig-Tran et al. 2013). Filter lobe and gill arch morphology may differ from pups to adults, and these differences may support shifts in diet or changes in flow regimes that occur during growth. Further investigation into the ontogeny of mobulid species may also aid efforts to scale bioinspired filter designs to different particle sizes and technical processes. Additional studies on feeding behavior would provide valuable information for understanding the functional constraints and selective pressures on filtering structures in different mobulid species. Although euphausiids appear to be the primary food of many mobulid species, gut content studies indicate that *M. birostris* and *M. tarapacana* also feed on fishes and squid (Rohner et al. 2017; Stewart et al. 2018). By examining species adapted to efficiently retain small prey particles, those targeting larger prey items, those that handle diverse particle shapes and materials, and those that filter high particle concentrations without clogging, we can identify specific design principles relevant to engineering. Understanding variation and function in nature can inform and drive innovative technological solutions, highlighting the value of a biology-first approach to bioinspiration (Cohen et al. 2014; Snell-Rood and Smirnoff 2023).

## Author contributions

The project was conceptualized by S.R.K.R., J.B.T., and E.M.W.P.T. Original scans were collected by S.R.K.R., J.B.T., and K.E.C. Data curation, formal analysis, and visualization were conducted by S.R.K.R., J.B.T., K.E.C., and L.H. Conceptualization and writing of the original draft were done by S.R.K.R. and J.B.T. Writing, reviewing, and editing were done by all co-authors. The project was supervised by E.M.W.P.T. and J.A.S.

## Supplementary Material

icaf142_Supplemental_File

## Data Availability

All scans are available at MorphoSource (https://www.morphosource.org/projects/000763113?locale=en). Code for generating the phylomorphospace, figures, and statistical analyses available in a Github repository (https://github.com/shirelkr/manta_formandfunction).

## Notes

From the symposium “Evolution to innovation: Bridging biology and engineering through bioinspired design” presented at the annual meeting of the Society for Integrative and Comparative Biology, January 3-7th , 2025.

## References

[bib1] Albert P . 2007. The flying mobulas of the Sea of Cortez. 28(1):Highlands, NJ: Underwater Naturalist.

[bib2] Bigelow AF , Schroeder WC. 1953. Bigelow and Schroeder's fishes of the Gulf of Maine. 3rd ed. Collette BB., Klein-MacPhee G. Washington, DC: Smithsonian Institution Press.

[bib3] Broeckhoven C , du Plessis A. 2022. Escaping the Labyrinth of Bioinspiration: biodiversity as Key to Successful Product Innovation. Adv Funct Materials. 32:2110235.

[bib4] Chao J , Cheng-Long YC, Chou H-H, Lin P-C, Chao R, Shih Y-T, Chien Y-C. 2020. 2020 Global Design Challenge Finalist: fine Particulate Matter Filters Inspired by Manta RaysAskNature. (https://asknature.org/innovation/rticulate-matter-filters-inspired-by-manta-rays/).

[bib5] Clark AS , San-Miguel A. 2021. A bioinspired, passive microfluidic lobe filtration system. Lab Chip. 21:3762–74. 34581374 10.1039/d1lc00449b PMC8486309

[bib6] Cohen YH , Reich Y, Greenberg S. 2014. Biomimetics: structure–function patterns approach. J Mech Des. 136.

[bib7] Couturier LIE , Marshall AD, Jaine FRA, Kashiwagi T, Pierce SJ, Townsend KA, Weeks SJ, Bennett MB, Richardson AJ. 2012. Biology, ecology and conservation of the Mobulidae. J Fish Biol. 80:1075–119. 22497374 10.1111/j.1095-8649.2012.03264.x

[bib8] Cuevas-Zimbrón E , Sosa-Nishizaki O, Pérez-Jiménez JC, O'Sullivan JB. 2013. An analysis of the feasibility of using caudal vertebrae for ageing the spinetail devilray, Mobula japanica (Müller and Henle, 1841). Environ Biol Fish. 96:907–14.

[bib9] Divi RV , Strother JA, Paig-Tran EWM. 2018. Manta rays feed using ricochet separation, a novel nonclogging filtration mechanism. Sci Adv. 4:eaat9533. 30263959 10.1126/sciadv.aat9533 PMC6157963

[bib10] Fedorov A , Beichel R, Kalpathy-Cramer J, Finet J, Fillion-Robin J-C, Pujol S, Bauer C, Jennings D, Fennessy F, Sonka M et al. 2012. 3D Slicer as an Image Computing Platform for the Quantitative Imaging Network. Magn Reson Imaging. 30:1323–41. 22770690 10.1016/j.mri.2012.05.001 PMC3466397

[bib11] Hamann L , Schreiber K, Hagenmeyer J, Eduardo S, Spanke T, Blanke A. 2023. Diversity of filter feeding and variations in cross-flow filtration of five ram-feeding fish species. Front Mar Sci. 10.

[bib12] Hu X , Yu L, Zhu Z, Bao F, Lin J, Tu C, Lin P. 2024. A self-cleaning micro-fluidic chip biospired by the filtering system of manta rays. Lab Chip. 24:3064–79. 38757493 10.1039/d4lc00039k

[bib13] Hughes GM , Morgan M. 1973. The Structure of Fish Gills in Relation to Their Respiratory Function. Biol Rev. 48:419–75.

[bib14] Kahane-Rapport SR , Paig-Tran EWM. 2023. Filtration in fishes. In: Fudge, Encyclopedia of Fish Physiology. Amsterdam, Netherlands: Elsevier.

[bib15] Kahane-Rapport SR , Teeple J, Liao JC, Paig-Tran EWM, Strother JA. 2025. Filter feeding in devil rays is highly sensitive to morphology. Proc R Soc B. 292:20242037. 10.1098/rspb.2024.2037 PMC11750363 39837516

[bib16] Kiyatake I , Ito K, Yoshii Y, Miyagawa Y, Kitadani Y, Nishida K. 2025. Insights Into the Reproduction and Maturity of the Spinetail Devil Ray (Mobula mobular). Zoo Biol. 189–196. 39821940 10.1002/zoo.21888

[bib46_857_195125] Klimley AP , Curtis TH, Johnston EM, Kock AA, Stevens GMW. 2024. A review of elasmobranch breaching behavior: why do sharks and rays propel themselves out of the water into the air?. Environ Biol Fish. 441–481.

[bib17] Mao X , Bischofberger I, Hosoi AE. 2024. Permeability–selectivity trade-off for a universal leaky channel inspired by mobula filters. Proc Natl Acad Sci USA. 121:e2410018121. 39586001 10.1073/pnas.2410018121 PMC11648657

[bib18] Marshall LE . 2019. Manta-inspired Robotic Platform and Filter Design for Mitigating Near-Shore Harmful Algal Blooms(Master's Thesis). Toledo (OH): University of Toledo(http://rave.ohiolink.edu/etdc/view?acc_num=toledo1556733016555221).

[bib19] Masangcay SI , Metillo E, Hayashizaki K, Tamada S, Nishida S. 2018. Feeding Habits of Mobula japanica Feeding Habits of Mobula japanica (Chondrichthyes, Mobulidae) in Butuan Bay, Mindanao Island, Philippines. Science Diliman. 30.24–44.

[bib20] Motta PJ , Maslanka M, Hueter RE, Davis RL, De L, Parra R, Mulvany SL, Habegger ML, Strother JA, Mara KR et al. 2010. Feeding anatomy, filter-feeding rate, and diet of whale sharks Rhincodon typus during surface ram filter feeding off the Yucatan Peninsula, Mexico. Zoology. 113:199–212. 20817493 10.1016/j.zool.2009.12.001

[bib21] Notarbartolo-Di-Sciara G . 1987. A revisionary study of the genus Mobula Rafinesque, 1810 (Chondrichthyes: mobulidae) with the description of a new species. Zool J Linn Soc. 91:1–91.

[bib22] Notarbartolo-di-Sciara G . 1988. Natural history of the rays of the genus Mobula in the Gulf of CaliforniaUS Fish and Wildlife Service Fishery Bulletin. (https://www-webofscience-com.lib-proxy.fullerton.edu/wos/woscc/full-record/WOS:A1988P107100003?SID=USW2EC0CE1a5Lc9wCr4ZPFeLaPKhK).

[bib23] Ormond R , Edwards A. 1987. Red Sea Fishes. In: Illumina Biological Content—Unstructured United Kingdom. Oxford, UK: Pergamon Press

[bib24] Paig-Tran EWM , Bizzarro JJ, Strother JA, Summers AP. 2011. Bottles as models: predicting the effects of varying swimming speed and morphology on size selectivity and filtering efficiency in fishes. J Exp Biol. 214:1643–54. 21525310 10.1242/jeb.048702

[bib25] Paig-Tran EWM , Kleinteich T, Summers AP. 2013. The filter pads and filtration mechanisms of the devil rays: variation at macro and microscopic scales. J Morphol. 274:1026–43. 23686500 10.1002/jmor.20160

[bib26] Paig-Tran EWM , Summers AP. 2014. Comparison of the structure and composition of the branchial filters in suspension feeding elasmobranchs. Anat Rec. 297:701–15. 10.1002/ar.22850 24443216

[bib27] Pardo SA , Kindsvater HK, Cuevas-Zimbrón E, Sosa-Nishizaki O, Pérez-Jiménez JC, Dulvy NK. 2016. Growth, productivity and relative extinction risk of a data-sparse devil ray. Sci Rep. 6:33745. 27658342 10.1038/srep33745 PMC5034314

[bib28] Poortvliet M , Olsen JL, Croll DA, Bernardi G, Newton K, Kollias S, O'Sullivan J, Fernando D, Stevens G, Galván Magaña F et al. 2015. A dated molecular phylogeny of manta and devil rays (Mobulidae) based on mitogenome and nuclear sequences. Mol Phylogenet Evol. 83:72–85. 25462995 10.1016/j.ympev.2014.10.012

[bib29] Porsiel N , Hernández S, Cordier D, Heidemeyer M. 2021. The devil is coming: feeding behavior of juvenile Munk’s devil rays (Mobula munkiana) in very shallow waters of Punta Descartes, Costa Rica. Rev Biol Trop. 69:S256–66.

[bib30] Rohner CA , Burgess KB, Rambahiniarison JM, Stewart JD, Ponzo A, Richardson AJ. 2017. Mobulid rays feed on euphausiids in the Bohol Sea. R Soc Open Sci. 4:161060. 28572998 10.1098/rsos.161060 PMC5451799

[bib31] Rojas A , Daniel S, Wright T. 2022. Youth Design Challenge 2022: The Filtration Pipes. (https://www.labxchange.org/library/items/lb:LabXchange:1519d94a:lx_case_study:1).

[bib32] Rubenstein DI , Koehl MAR. 1977. The mechanisms of filter feeding: some theoretical considerations. Am Nat. 111:981–94.

[bib33] Sampson L , Galván-Magaña F, Silva-Dávila RD, Aguíñiga-García S, O'Sullivan JB. 2010. Diet and trophic position of the devil rays Mobula thurstoni and Mobula japanica as inferred from stable isotope analysis. J Mar Biol Ass. 90:969–76.

[bib35] Sanderson SL , Cheer AY, Goodrich JS, Graziano JD, Callan WT. 2001. Crossflow filtration in suspension-feeding fishes. Nature. 412:439–41. 11473318 10.1038/35086574

[bib34] Sanderson SL . 2024. Particle separation mechanisms in suspension-feeding fishes: key questions and future directions. Front Mar Sci. 11.

[bib36] Sankrityayan P , Biswas S. 2022. Plastic Filtration and Decomposition According to Ricochet Filtering Mechanism Using Ideonella sakaiensis. Front Mar Sci. 9.

[bib37] Schneider CA , Rasband WS, Eliceiri KW. 2012. NIH Image to ImageJ: 25 years of image analysis. Nat Methods. 9:671–5. 22930834 10.1038/nmeth.2089 PMC5554542

[bib38] Smith JC , Sanderson SL. 2008. Intra-Oral Flow Patterns and Speeds in a Suspension-Feeding Fish With Gill Rakers Removed Versus Intact. Biol Bull. 215:309–18. 19098151 10.2307/25470714

[bib39] Smith JLB . 1943. Interesting New Fishes of Three Genera New to South Africa, with a Note on Mobula Diabolus (shaw). Trans R Soc South Africa. 30:67–77.

[bib40] Snell-Rood EC , Smirnoff D. 2023. Biology for biomimetics I: function as an interdisciplinary bridge in bio-inspired design. Bioinspir Biomim. 18:052001. 10.1088/1748-3190/ace5fb 37429293

[bib41] Stewart JD , Jaine FRA, Armstrong AJ, Armstrong AO, Bennett MB, Burgess KB, Couturier LIE, Croll DA, Cronin MR, Deakos MH et al. 2018. Research Priorities to Support Effective Manta and Devil Ray Conservation. Front Mar Sci. 5.

[bib42] van der Meer S , Miyuki FN, Fernald J, Shieh D, Lange A. 2019. 2019 Global Design Challenge Finalist: plastic Pollution Filter Inspired by the Manta Ray and Basking SharkAskNature. (https://asknature.org/innovation/plastic-filtering-device-inspired-by-the-manta-ray-and-basking-shark/).

[bib43] White WT , Corrigan S, Yang L, Henderson AC, Bazinet AL, Swofford DL, Naylor GJP. 2018. Phylogeny of the manta and devilrays (Chondrichthyes: mobulidae), with an updated taxonomic arrangement for the family. Zool J Linn Soc. 182:50–75.

[bib44] White WT , Giles J, Dharmadi PIC. 2006. Data on the bycatch fishery and reproductive biology of mobulid rays (Myliobatiformes) in Indonesia. Fish Res. 82:65–73.

[bib45] Wilson JM , Laurent P. 2002. Fish gill morphology: inside out. J Exp Zool. 293:192–213. 12115897 10.1002/jez.10124

