# Supplementary material for: Form and Function in Mobulids: A Comparative Analysis of Filter Morphology With Bioinspiration Applications
Source: Integr Comp Biol. 2025 Aug 1;65(6):1576–600. doi: 10.1093/icb/icaf142 (PMC12690469; doi:10.1093/icb/icaf142)
Supplement: icaf142_Supplemental_File [file icaf142_supplemental_file.docx]

Supplemental Table 1. Number of measurements sampled from the posterior side of the arch.

| **Measurement** | **Position** |  |  |  | **Specimen** | |  |  |  |
| --- | --- | --- | --- | --- | --- | --- | --- | --- | --- |
|  |  | *M. birostris* | *M. hypostoma* | *M. kuhlii* | *M.kuhlii (cf. eregoodoo)* | *M. mobular* | *M. mobular (cf. japanica)* | *M. munkiana* | *M. thurstoni* |
| Primary Pore Span(PPS) | Proximal | n = 6 | n = 5 | n = 5 | n = 4 | n = 5 | n = 5 | n = 4 | n = 5 |
|  | Middle | NA | n = 5 | n = 5 | n = 4 | n = 5 | n = 5 | n = 5 | n = 5 |
|  | Distal | NA | n = 5 | n = 5 | n = 4 | n = 5 | n = 5 | n = 5 | n = 5 |
| Primary Pore Width(PPW) | Proximal | n = 6 | n = 5 | n = 5 | n = 4 | NA | n = 5 | n = 5 | n = 4 |
|  | Middle | NA | n = 5 | n = 5 | n = 4 | NA | n = 5 | n = 5 | n = 4 |
|  | Distal | NA | n = 5 | n = 5 | n = 4 | NA | n = 5 | n = 5 | n = 2 |
| Primary Pore Period(PPP) | Proximal | n = 6 | n = 5 | n = 5 | n = 4 | n = 5 | n = 5 | n = 5 | n = 5 |
|  | Middle | NA | n = 5 | n = 5 | n = 4 | n = 5 | n = 5 | n = 5 | n = 5 |
|  | Distal | NA | n = 5 | n = 5 | n = 4 | n = 5 | n = 5 | n = 5 | n = 5 |
| Primary Pore Length (PPL) | Proximal | n = 6 | n = 5 | n = 4 | n = 4 | NA | n = 5 | n = 4 | n = 5 |
|  | Middle | NA | n = 5 | n = 5 | n = 4 | NA | n = 5 | n = 5 | n = 5 |
|  | Distal | NA | n = 5 | n = 5 | n = 4 | NA | n = 5 | n = 5 | n = 5 |
| Lobe Angle (LA) | Proximal | n = 6 | n = 5 | n = 4 | n = 4 | n = 5 | n = 5 | n = 5 | n = 5 |
|  | Middle | NA | n = 5 | n = 5 | n = 4 | n = 3 | n = 5 | n = 5 | n = 5 |
|  | Distal | NA | n = 5 | n = 5 | n = 4 | n = 3 | n = 5 | n = 5 | n = 5 |
| Lobe Radius of Curvature (LRC) | Proximal | n = 5 | n = 4 | n = 5 | n = 2 | n = 5 | n = 5 | n = 5 | n = 5 |
|  | Middle | NA | n = 5 | n = 5 | n = 4 | n = 5 | n = 4 | n = 5 | n = 5 |
|  | Distal | NA | n = 5 | n = 5 | n = 4 | n = 5 | n = 5 | n = 5 | n = 5 |
| Secondary Pore Width (SPW) | Proximal | NA | n = 5 | n = 4 | n = 3 | NA | n = 5 | n = 4 | NA |
|  | Middle | NA | n = 5 | n = 5 | n = 4 | NA | n = 5 | n = 5 | NA |
|  | Distal | NA | n = 5 | n = 5 | n = 3 | NA | n = 5 | n = 4 | NA |
| Secondary Pore Height (SPH) | - | NA | n = 5 | n = 5 | n = 4 | NA | n = 5 | n = 5 | NA |
| Secondary Pore Length (SPH) | - | NA | n = 5 | n = 4 | n = 4 | NA | n = 5 | n = 5 | NA |
| Plate Radius of curvature (PRC) | - | NA | n = 5 | n = 5 | n = 2 | NA | n = 5 | n = 5 | NA |

Supplemental Table 2. Number of measurements sampled from the anterior side of the ceratobranchial arch.

| **Measurement** | **Position** |  |  | **Specimen** |  |  |
| --- | --- | --- | --- | --- | --- | --- |
|  |  | *M. mobular* | *M. mobular (cf. japanica)* | *M. munkiana* | *M. tarapacana* | *M. thurstoni* |
| Primary Pore Span(PPW) | Proximal | NA | n = 1 | NA | n = 9 | n = 4 |
|  | Middle | n = 5 | n = 1 | NA | NA | n = 1 |
|  | Distal | n = 2 | n = 1 | n = 6 | NA | n = 4 |
| Primary Pore Width (PPH) | Proximal | NA | n = 1 | NA | n = 9 | n = 4 |
|  | Middle | NA | n = 1 | NA | NA | n = 1 |
|  | Distal | NA | n = 1 | n = 6 | NA | n = 3 |
| Primary Pore Period(PPP) | Proximal | NA | n = 1 | NA | n = 9 | n = 4 |
|  | Middle | n = 5 | n = 1 | NA | NA | n = 1 |
|  | Distal | n = 2 | n = 1 | n = 6 | NA | n = 4 |
| Primary Pore Length (PPL) | Proximal | NA | NA | NA | n = 9 | n = 4 |
|  | Middle | NA | NA | NA | NA | n = 1 |
|  | Distal | NA | NA | n = 6 | NA | n = 4 |
| Lobe Angle (LA) | Proximal | NA | n = 1 | NA | n = 9 | n = 4 |
|  | Middle | n = 5 | n = 1 | NA | NA | n = 1 |
|  | Distal | n = 1 | n = 1 | n = 6 | NA | n = 2 |
| Lobe Radius of Curvature (LRC) | Proximal | NA | n = 1 | NA | n = 6 | n = 4 |
|  | Middle | n = 5 | n = 1 | NA | NA | n = 1 |
|  | Distal | n = 2 | n = 1 | n = 6 | NA | n = 4 |
| Secondary Pore Width (SPW) | Proximal | NA | n = 1 | NA | n = 2 | NA |
|  | Middle | NA | n = 1 | n = 2 | NA | NA |
|  | Distal | NA | NA | n = 2 | NA | NA |
| Secondary Pore Height (SPH) | - | NA | n = 1 | n = 2 | n = 3 | NA |
